# Polymer Nanocomposites of Selenium Biofabricated Using Fungi

**DOI:** 10.3390/molecules26123657

**Published:** 2021-06-15

**Authors:** Olga Tsivileva, Alexander Pozdnyakov, Anastasiya Ivanova

**Affiliations:** 1 Institute of Biochemistry and Physiology of Plants and Microorganisms, Russian Academy of Sciences, 13 Prospekt Entuziastov, 410049 Saratov, Russia; 2 A.E. Favorsky Irkutsk Institute of Chemistry, Siberian Branch of the Russian Academy of Sciences, 1 Favorsky St., 664033 Irkutsk, Russia; pozdnyakov@irioch.irk.ru (A.P.); ivanovaAA-1964@yandex.ru (A.I.)

**Keywords:** polymer nanocomposites, selenium nanoparticles, selenium-containing biocomposites, green synthesis, fungi, mushrooms, mechanism

## Abstract

Nanoparticle-reinforced polymer-based materials effectively combine the functional properties of polymers and unique characteristic features of NPs. Biopolymers have attained great attention, with perspective multifunctional and high-performance nanocomposites exhibiting a low environmental impact with unique properties, being abundantly available, renewable, and eco-friendly. Nanocomposites of biopolymers are termed green biocomposites. Different biocomposites are reported with numerous inorganic nanofillers, which include selenium. Selenium is a micronutrient that can potentially be used in the prevention and treatment of diseases and has been extensively studied for its biological activity. SeNPs have attracted increasing attention due to their high bioavailability, low toxicity, and novel therapeutic properties. One of the best routes to take advantage of SeNPs’ properties is by mixing these NPs with polymers to obtain nanocomposites with functionalities associated with the NPs together with the main characteristics of the polymer matrix. These nanocomposite materials have markedly improved properties achieved at low SeNP concentrations. Composites based on polysaccharides, including fungal beta-glucans, are bioactive, biocompatible, biodegradable, and have exhibited an innovative potential. Mushrooms meet certain obvious requirements for the green entity applied to the SeNP manufacturing. Fungal-matrixed selenium nanoparticles are a new promising biocomposite material. This review aims to give a summary of what is known by now about the mycosynthesized selenium polymeric nanocomposites with the impact on fungal-assisted manufactured ones, the mechanisms of the involved processes at the chemical reaction level, and problems and challenges posed in this area.

## 1. Introduction

Fungal-assisted fabrication of nanoparticles (NPs), with their broad set of applications, has attracted the attention of scientists to a novel area of research, myconanotechnology [1], which manipulates the matter in a nanoscale order and includes mainly the synthesis protocols of NPs using fungal biomass and metabolites. Selenium is an essential microelement in every living organism, and in humans, it encourages the immune system to perform properly and exhibits powerful anticancer and antimicrobial effects [2]. More ecologically safe and beneficial approaches to obtaining the Se-based products are the current challenge. By combining nanotechnology with the intrinsic biological activity of selenium, unexpectedly efficient tools for possible applications of selenium-containing NPs (SeNPs) could be identified because of their appealing physicochemical and functional properties [3,4]. However, the SeNPs were prone to aggregation into clusters in liquid media, leading to a much lower bioactivity and bioavailability. A solution appeared to be found, as the modification of the SeNP surface with polymers can lead to dispersed particles in the polymer matrix and the occurrence of nanocomposites.

Polymer-based particulate systems have found widespread applications in different areas of research as well as everyday life [5]. Polymeric nanomaterials are promising for the development of substances for magnetic resonance tomography, efficient media for magnetic-controlled transfer of drugs, agents for the treatment of malignant tumors, and systems of targeted delivery of contrasting and medicinal agents [6]. Polymer nanocomposites have been a focus of attention, particularly because of their enhanced physicochemical, engineering [7], and drug-conjugating [8] properties. Such nanocomposites have found their extensive applications in diverse fields ranging from food packaging [9] and the environment [10,11], including water and wastewater treatment [12] to medicine [13,14]. Today, myconanotechnology has broadened the scope of polymeric materials application by introducing the polymer element-containing nanocomposites, where the element may be selenium. In this way, the goal of dramatically enhanced performance of both SeNPs and the polymer matrix itself could be achieved. Comparative studies of the chemically and biologically synthesized polymeric composites comprising SeNPs would contribute to the scientific foundations of manufacturing and implementing the innovative agents under question. Both the mentioned approaches have deserved a lot of attention and are discussed in this review, with the focus on mycosynthesis offering countless possibilities.

Even though several reviews related to the natural production of valuable NPs by means of microorganisms were published in 2014 [3,15] and very recently [16], the overwhelming majority have been devoted to metallic NPs [17,18,19]. The pieces of information on the mechanisms behind the bio-mediated synthesis of SeNPs to yield their biocomposites have not been compiled yet. The attempt to somehow fill this gap in the review would allow the production of selenium-based polymer nanocomposites mediated by medicinal and edible fungal cultures to be put into practice. Moreover, several low-molecular-mass Se-supplying compounds along with polymeric components, both synthetic and natural ones, for the Se-nanocomposites synthesis procedures and some chemical aspects of their formation, advantages, and limitations, as well as challenges encountered, are considered.

## 2. On the Definitions of Nanocomposites

A composite is a generally recognized material composed of one to several different materials or polymers with a marked difference in chemical or physical properties. Composites combine different materials in a single device [20]. Composites are used to produce customized properties through enhancing or imparting certain features that cannot be demonstrated by particular homogeneous substances [21]. Composites are capable of having their properties tailored by a series of variables, as the type, concentration, size, and shape of the constituents.

Nanomaterials are materials that have structural nanosized components. For the last one or two decades, nanomaterials have been successfully applied to various and numerous areas such as medicine, biology, catalysis, sensing, etc., owing to their unique mechanical, electrical, optical, and magnetic properties when compared with conventional materials. In recent years, nanotechnology merging with material sciences has resulted in the development of nanocomposite materials with highly enhanced thermal, catalytic, electrical, optical, and mechanical properties compared to the individual components. One of the approaches was to introduce various kinds of fillers into the polymeric matrix to provide reinforcing filler composites. These complex materials are generally prepared by the incorporation of inorganic fillers into polymer matrices [7] using various processing techniques. The science of particle reinforced composites has shown that the size of filler materials has a profound effect on the resulting properties of the composite. This is because surface interactions with the matrix, adhesion, particle motion, dispersion, bonding, etc., depend a great deal on the size of the filler particle. Because some of these effects increase with a decrease in filler size, they have a more profound impact on the properties at the nanoscale. Nanoscale fillers possess very large surface-to-volume ratio, and because properties like catalytic and chemical reactivity and others depend on the nature of the interface, these properties change dramatically. When a polymer matrix is combined with reinforcement materials having at least one of its dimensions in the nanoscale, a 3D structure is formed called polymer nanocomposite material. The development of this technology has enthused researchers to explore promising nanocomposites with a large surface-to-volume ratio compared to their bulk material (metal or non-metal). Polymer–nonmetallic composites can be fabricated by the incorporation of nonmetal fillers inside the polymer matrix or as coatings in the polymer surface, producing the so-called polymer–nonmetal nanocomposites.

Eco-friendly and green chemistry approaches are appreciated to avoid possible environmental contamination. Such environmentally friendly, renewable polymer composites are called green polymer (nano)composites, the major attractions of which are sustainability and biodegradability along with impressive physicochemical properties. When polymeric composites contain specific biological phases, they are defined as biocomposites [22]. Biogenic polymer nanocomposites have appeared owing to the fact that the above manufacturing processes could be performed by microorganisms including fungal cultures. In particular, mushroom-based microbial biosynthesis is emerging as a novel technique for the production of diverse forms of NPs, as the technique is economic, non-laborious, and most importantly eco-friendly.

## 3. Fungal Features to Be Recruited in Se-Nanomaterial Synthesis

In general, when conventional physical and chemical methods of nanomaterial synthesis run into bottlenecks, researchers have begun to pursue a milder and more eco-friendly approach [23]. Along with Se-nanomaterials fabricated by physical synthesis or chemical reduction of inorganic salts, research has focused on developing a nature-derived alternative production method [16]. The use of pathogenic microbes is not advisable for nanomaterial synthesis. Restrictions in further applications of the produced NPs should be remembered, involving human, animal, or other living organism contacts in the environment [24]. NP synthesis with eukaryotic microorganisms has emerged as a better alternative to prokaryotes due to their high intracellular uptake capability, ability to synthesize NPs with different chemical compositions, ability to produce a large number of metabolites per unit biomass, and easy biomass handling at the laboratory scale [25]. A reliable and nontoxic method to manufacture SeNPs applicable to the food and biomedical fields can be realized through biogenic procedures using fungi [26].

Fungi are eukaryotic microorganisms preferred over other organisms for the large-scale production of NPs [27]. Fungal biomass normally grows faster than that of bacteria [28], and fungi keep on growing even after the formation of NPs. The development of mycelium with its large surface area for interactions is accompanied by the accumulation of a variety of fungal biopolymers offering numerous reaction sites for the formation of NPs and their protection/entrapment within the shell structure [29]. Live fungus and isolated fungal-cell fractions frequently show an equal efficiency of NPs production and properties [24]. It is evident that the accumulated NPs trigger several physiological and biochemical changes in the microbes; however, fungi can tolerate even a toxic NP material (as heavy metal) in the reaction medium at a level greatly exceeding the accepted permissible limit in a contaminated environment [30]. In the presence of fungi, many types of NPs could form on the surface of mycelia and even a transformation monitored visually by a change in color of the fungal biomass, e.g., pink to red coloring of medium or mycelium caused by visually observable elemental selenium deposition [31]. Monitoring of color change to different degrees of orange or red color has been repeatedly used to assess the SeNPs bioproduction [32,33].

There are several reports available on the NPs’ synthesis using fungi. Major work has been done with silver NPs produced by fungi extracellularly or intracellularly since 2001 [34,35,36,37]. Ag-nanostructures can start from different extracts featured by the plentiful and diversified metabolites [38], including extracts from mushrooms. Vamanu et al. [39] described a mushroom-extract-based green AgNPs synthesis process mediated by *Lactarius deliciosus*. Moreover, it was the first report to characterize the effect of AgNPs against some bacteria as a part of the intestinal microbiota, in terms of both metabolic and biodiversity changes. In particular, a synergistic antibacterial activity against various pathogenic microorganisms was established [39].

Biosynthesis of gold NPs from fungi was reviewed in 2015 [40] and has been further developed [41,42]. The works [17,27,43,44,45] focus on the synthesis of metal NPs of varying sizes and shapes from the diverse fungal species. The authors also discuss the metal NPs’ potential bio-prospective applications. Metal oxide-based NPs were also studied. Salunke et al. [46] suggested the role of the *Saccharomyces cerevisiae* cell wall composed of chitin, glucan, mannoprotein, other proteins, etc., in Mn(IV) oxide NPs’ synthesis and stabilization. Many other mycosynthesized metal-oxide-based NPs have been explored within this promising field of research [47]. Filamentous fungi were reported for SeNPs synthesis. *Aspergillus terreus* and *A. oryzae* were shown to biosynthesize Se nanoparticles [48,49]. *A. terreus* cell-free filtrate produced polydisperse Se nanoparticles of the size 10–100 nm. Mosallam et al. employed a distinct approach to SeNPs mycosynthesis by exposing the cell-free filtrate to Se-containing ions in the presence of gamma rays to maximize NP production [49].

Synthesis of NPs using mushrooms is more advantageous in terms of culturing and handling, since the latter are nontoxic, handy, and amenable [50,51]. Mushrooms are part of fungal biota characterized by wonder [52]. Mushrooms are macrofungi with a different form of distinctive fruiting bodies; they are large enough to be seen with the naked eye [53], with mycelium being a vegetative phase and fruiting bodies being a reproductive phase. Mushrooms can be classified in basidiomycetes and ascomycetes based on the development patterns in the life cycle [54,55]. Edible and medicinal mushrooms have long been known by humanity as valuable food, decomposers in ecosystems, and producents of unique complex of biologically active substances. Many studies have presented evidence on the correlation between the consumption of mushrooms and human health. The effect of six edible wild mushroom species (*Boletus edulis, B. pinophilus, B. aureus, Armillaria mellea, Lactarius piperatus, Pleurotus eryngii*) accompanied by a positive control (*P. djamor*) and negative control (*Amanita rubescens*) on the microbiota was explored to identify the microbial fingerprint in the descending colon [56]. The consumption of these mushrooms occurred, followed by a positive shift in the amount of bifidobacteria, lactobacilli, and entrobacteriacee capable of sustaining the balance between hosts and symbiotic microbes. Mushrooms are rich in powerful reducing agents, phenolic compounds [57]. To determine the effect on free radicals, lipid peroxidation, and reducing power in ten commonly consumed mushroom species, the content of polyphenol carboxylic acids was assayed [58]. These acids’ presence, along with other bioactive molecules such as flavonoids, carotenoid compounds, etc., was correlated with the antioxidant capacity expressed in vitro.

For fungal-assisted production of SeNPs, the type of the green entity and its composition are crucial [16]. Macroscopic basidiomycetes meet certain obvious requirements for the non-toxic green entity applied to the SeNP manufacturing. The advantages of using mushrooms for nanomaterials bioproduction are as follows: (1) mushrooms are capable of growing in the form of mycelial biomass in submerged cultures; (2) the overwhelming majority of biotechnologically applied mushrooms are cultivable commercially under the same circumstances, yielding identical products to ascertain reproducibility; (3) mycelia are easily propagated vegetatively and thus keep to one clone, and the genetic and biochemical consistency can be checked after a considerable time [52]. The broad spectrum and diversity in mushrooms belonging to different ecological and taxonomic groups could lead to an exciting potentiality and interdisciplinarity of myconanotechnology.

Therefore, after carefully examining the microbe-assisted syntheses of NPs, it can be concluded that biological entities applicable to SeNP biofabrication could be mushrooms, considered frequently to be ideal candidates for nanomaterial bioproduction. Primary and secondary fungal metabolites are capable of readily reducing the chemical elements in compounds to cause nanoparticles with definite size and shape in controlled non-hazardous processes. Information on the chemical forms of selenium moiety in edible and medicinal mushrooms grows increasingly. This database indicates that the Se-containing compounds identified in fruit bodies include naturally occurring organoselenium amino acids, namely selenomethionine SeMet, selenocysteine SeCys with its dimer selenocystine (SeCys)_2_, Se-methylseleno-*L*-cysteine MeSeCys, few inorganic selenium species [59], and separate analytically unidentified selenium chemical forms, whose proportions vary widely [60]. To enhance the fortification of the submerged mycelia with selenium as selenoaminoacidic species, the optimization of sodium selenite level in liquid medium seems feasible. Thus, the concentration of MeSeCys, a seleno-amino acid with strong anticancer activity, was increased from virtually underdetectable quantities to 120 µg/g recalculated to dry weight when a nutrient medium was enriched with selenium [61]. *Agaricus bisporus* grown on the Se-irrigated compost provided much higher levels of SeCys (detected as its dimmer) compared to SeMet and MeSeCys, which contributed most significantly to the proportion of selenoamino acids per g protein, i.e., to the greater content of selenoamino acids as selenoproteins [62]. Another mushroom, *Lentinula edodes,* cultured in selenite-fortified media was capable of incorporating mainly the SeMet form of selenium into the fungal proteins [63]. However, in spite of the increasing moieties of both SeMet and selenium in the shiitake biomass with growth, the proportion of Se accumulated in the form of selenoamino acid decreased. Moreover, at a relatively high selenium level in the medium, the SeCys content of mycelium was not directly dependent on that Se-level and remained essentially unchanged. A considerable portion, nearly 50%, of selenium introduced in the form of an inorganic source of the culture of *Hericium erinaceus* (lion’s mane mushroom) was biotransformed into SeMet and MeSeCys [64]. Three Se-adenosyl-comprising species were detected as Se-methyl-5-selenoadenosine, Se-methyl-5-selenoadenosine-Se-oxide, and Se-dimethyl-5-selenonium-adenosine, unreported previously.

To develop bioproduction of Se-nanomaterial by means of mushroom supplemented with Na_2_SeO_3_ or Na_2_SeO_4_, the observations of Se^0^ form in the components of fungal culture attracted the attention of many researchers in the 21st century. Submerged mycelium of shiitake mushroom was capable of accumulating Se efficiently from liquid nutrient media. Different biosamples of selenized biomass were compared [65]. Selenium speciation in Se-supplied mycelial cultures provided evidence that a large portion of selenium presented in zero and four oxidation states. Selenite oxyanions in nutrient media caused a response in *Phanerochaete chrysosporium*, a mushroom highly sensitive to selenite, manifested in inhibition of its growth and substrate intake at a level of 10 mg/L. *P. chrysosporium,* like other mushrooms, was capable of reducing Se(IV), but not Se(VI), oxyanion to yield elemental selenium [66]. In the course of experiments with *Agrocybe aegerita*, *Hericium erinaceus*, and *Ganoderma lucidum*, the latter mushroom was observed to produce fruit bodies on a selenized substrate with up to 0.8 mM Se. Moreover, high selenium content in terms of total Se accompanied by pigmentation changes of fruiting bodies, probably owing to elemental selenium pull, was demonstrated [67]. Biotechnologically important mushrooms fortified with inorganic Se, such as *Ganoderma lucidum*, *Pleurotus ostreatus*, *Pleurotus eryngii*, *Pleurotus pulmonarius*, *Flammulina velutipes*, *Ganoderma applanatum*, *Lenzites betulinus*, and *Trametes hirsuta,* were explored [68] with respect to their morpho-physiological characteristics as well as biological activities. During cultivation on selenite-enriched medium, the appearance of mycelium of brick-red color with significant morphological and ultrastructural changes in comparison with the control was observed. Hyphal density was lower, the cell wall was thick with more expressed extracellular matrix, septa were abundant, and branch frequency and occurrence of clamp-connections were rare. Cytological analysis demonstrated that the majority of selenium was accumulated in cell membrane and vacuoles, while changes taking place in a cell wall were insignificant [68].

A fairly large amount of works have focused on the effect of different inorganic Se species on morphological and ultrastructural features of mycelia. At the elevated concentrations of Se(IV) oxyanions in the liquid nutrient medium of shiitake mushroom, the excessive selenium was biotransformed to Se^0^ via a reduction process [69]. Explorations of the oyster mushroom revealed that the elevated sodium selenite level resulted initially in total selenium accumulation in *Pleurotus ostreatus* mycelium, and then, at a suppressed growth phase, in Se(IV) reduction to amorphous Se^0^, thus imparting a reddish color to both biomass and medium [31,70]. The microstructure of *P. ostreatus* hyphae appeared to be dependent on selenium content in a liquid medium. Electron-dense moieties visible in both the Se-enriched and non-Se-enriched specimen were characterized [71] as proteinaceous bodies, the more so lipidic bodies were extracted in the course of sampling for TEM. Protein bodies’ quantity increased in the presence of selenium, with the changes in their morphology, pigmentation, and dimensions being only slight. Resulting from the oxyanionic Se reduction, amorphous Se^0^ occurred noticeably around the bodies [71]. Abiotic stress caused by inorganic Se(IV) induced a severe response in mushroom cultures manifested among others in ultrastructural features of hyphae and spores, e.g., in *Ganoderma lucidum* [72]. A number of studies described the selenium distribution over cellular compartments, in particular, polysaccharide moieties of fungal cell walls. Oyster mushroom submerged biomass enriched in Se was studied in respect to this microelement incorporation in mycelium from a liquid broth [73]. The polysaccharide fraction of selenized mycelia was treated with Tris-HCl or alternatively with chitinolytic enzyme. An increased proportion of low-molecular-mass substances in treated specimens was found in chitinase-assisted assays owing to the polysaccharides’ degradation in the enzymatic process. Thus, the confirmation of selenium binding to the chitin-containing polysaccharidic pull in the fungal cell wall was obtained [73]. In *Phanerochaete chrysosporium*, fungal pellets’ size diminution, compaction, and smoothness were induced by sodium selenite [66]. Analysis of *P. chrysosporium* mycelia with TEM, EELS, and a 3D reconstruction allowed a visualization of SeNPs bioproduced intracellularly. Therefore, mycelia of a lot of mushrooms studied at submerged culture conditions are appropriate sources of selenium, the concentration of which could be as high as ten percent of its external level in the Se(IV)-supplied medium, e.g., 62.5% for oyster mushroom [74]. Furthermore, Se content of mycelium could be several times higher than that of fruiting bodies.

**Figure 1:**
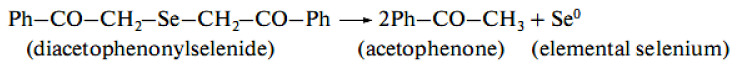
Scheme of DAPS-25 biotransformation.

The overwhelming majority of the published works on the Se-fortified fungal cultures have focused on Se chemical species exclusively in the form of the inorganic salts Na_2_SeO_3_ and Na_2_SeO_4_. A great deal of accumulated data indicate a highly important, if not decisive, role of Se chemical form played in its bioavailability and biotechnological applicability [75]. An extending amount of research provides evidence in favor of dramatically different biological consequences induced by inorganic and organic Se-agents [76,77]. The favorable chemical and biological profile of synthetic organoselenium compounds warrants their recognition as a promising option for fortification purposes. The Se-supplying substance should reasonably be of organoselenium nature, a prominent example being the substance 1,5-diphenyl-3-selenopentanedione-1,5 (DAPS-25; Selenoline, Selenobel, extra chemical names are diacetophenonylselenide, bis(benzoylmethyl)selenide) [78]. It is very low-toxic (compared to, e.g., selenites) at physiological concentrations, but has very efficiently been applied, over the last decades, to compensate for a selenium deficiency in various organisms and to prevent and treat infectious diseases. When the oyster mushroom and filamentous fungi (*Aspergillus niger*, *Chaetomium globosum*, and *Trichoderma viride*) were grown on wheat agar supplemented with organic selenide, the zero-valent-Se liberation was visually observable as deposition on the mycelium. A reddish mycelium coloring was especially pronounced for *Pleurotus ostreatus* [31]. Figure 1 depicts a scheme of selenium in zero oxidation state appearance in the above cases. This decay process does occur in the presence of fungal biomass.

The profound red pigmentation of biomass that resulted from diacetophenonyl selenide decomposition by the mushroom *Lentinula edodes* (shiitake) followed by the accumulation of elemental selenium in fungal culture was revealed. DAPS-25 biotransformation during the growth of this basidiomycete under the liquid-phase and solid-phase culture conditions has been studied [79]. As evidenced from the biomimetic chemical reaction, the data of X-ray diffraction, and gas-chromatography with mass-spectrometric analyses, the submerged shiitake mushroom culture was capable of decomposing the organoselenium agent to yield a red allotropic modification of elemental selenium. The process of elemental selenium elimination was followed by its precipitation onto gyphae [80].

In contact with a baker’s yeast *Saccharomyces cerevisiae* culture in different media, DAPS-25 was reduced to acetophenone and selenium nano- and micro-particles [81]. By means of high-performance liquid chromatography (HPLC) analysis of the benzene extracts of the experimental solution apparently containing the yeast-synthesized elemental selenium along with the residual organic selenide DAPS-25, the latter starting preparation was not found in the final reaction mixture. TEM discovered the formation of spherical nano- and microparticles 50 to 400 nm in size.

Within the framework of more recent studies, the growth parameters of more than twenty strains of xylotrophic basidiomycetes belonging to eight genera, 13 species on liquid media enriched with selenium in organic form were studied, and the effect of 1,5-diphenyl-3-selenopentanedione-1,5 within a wide concentration range (1·10^−4^ − 1·10^−8^ mol/L) on the mycelial growth was observed. The culture liquids of the fungal species under study were successfully tested for their reducing and stabilizing properties toward organic selenide and elemental selenium, respectively [82]. In doing so, the mycosynthesized Se-containg bionanocomposites were manufactured and tested for biological activity.

## 4. Synthesis and Mycosynthesis of Polymer Nanocomposites

### 4.1. Macromolecular Building Blocks for Nanocomposites

#### 4.1.1. Chemically Synthesized Polymer Matrices

Possession of inimitable physical, chemical, and biological properties of nanoparticles strongly depends on the synthetic route, reaction conditions, and type of reducing and/or stabilizing agent used in the reaction medium [83,84]. Synthetic organic polymers can serve as capping agents and increase the stability of NPs by covering the particle surface [85,86]. In these polymer nanocomposites, NPs have been associated with polymers such as polyvinyl alcohol [87,88], poly(methyl methacrylate) [89,90], polyurethanes [91,92,93], polylactic acid [94,95], polytetrafluoroethylene [96], polysiloxanes [97], and poly(lactic-*co*-glycolic acid) [98]. In the research [99], uniform, stable, amorphous SeNPs were synthesized and additionally immobilized within spherical poly(lactic-*co*-glycolic acid) particles. These particles were used to coat bioactive glass-based scaffolds synthesized by the foam replica method. Poly(2-oxazoline)- and poly(2-ethyl-2-oxazoline)-coated particles were explored by Wilson et al. [5]. The strategies of poly(2-oxazoline) or its derivative grafting to inorganic particles allowed the preparation of core-shell hybrid NPs, e.g., silica NPs were covalently grafted with a poly(2-ethyl-2-oxazoline) shell. Poly(2-ethyl-2-oxazoline) with a polymerization degree of 20 and 38 was synthesized and subsequently end-functionalized with a triethoxysilyl linker for grafting to SiO_2_ NPs (7, 31, and 152 nm) [5].

Numerous N-containing polymer classes are biodegradable and biocompatible and could be used to endow systems with desired properties. The attention to triazole derivatives is attracted, besides the theoretical interest, by their wide application in various fields, such as medicine, technique [100], and agriculture [101,102], where the integrated 1,2,4-triazole-containing fungicidal preparations serve as not only plant protectants, but also stimulants with plant growth regulatory activity [103]. To form synthetic polymer nanocomposites, SeNPs could also be associated with poly(vinyl pyrrolidone) [104,105,106,107]. The many scientific works published are a testimony to the role of poly(vinyl pyrrolidone) as a biodegradable polymer from a set of stabilizing agents applied to avoid agglomeration during the preparation of NPs [108]. Poly(vinyl pyrrolidone) as itself is frequently implemented in the synthesis of nanoparticles [83]. This polymer is among the commonly used coating agents or stabilizers like poly(vinyl alcohol), poly(ethylene glycol), citrate, etc., employed to prevent aggregation of nanoparticles synthesized through chemical reduction methods [109]. Interest in poly(vinyl pyrrolidone) application as a capping/reducing/nucleating agent is caused by its non-toxicity, good biodegradability, and many useful physicochemical properties [107,110].

Chemical polyfunctionality of vinyl derivative of 1,2,4-triazole provides wide opportunities for its use in the electrophilic addition, complexation, and polymerization reactions. The introduction of the nitrogen-containing heterocyclic 1-vinyl-1,2,4-triazole provides (co)polymers with solubility, including solubility in water, biocompatibility, chemical resistance, thermal stability, and complexation ability [111]. Homo- and copolymers of 1-vinyl-1,2,4-triazole are promising for the development of sorbents of noble metal ions [111,112], effective flocculants for food-industry applications [113], organic semiconductors and fuel cell membranes [114], and chelating polymer ligands [115]. Owing to their ligand properties, poly(1-vinyl-1,2,4-triazole) and its derivatives offer promise as the stabilizing matrices of metal nanoparticles of hybrid nanocomposites [116]. The growth-promoting effect of the chemically synthesized element-containing vinyltriazole-based polymers in respect to mushrooms has been elucidated [117]. High potentiality of using selenium-containing nanocomposites based both on 1-vinyl-1,2,4-triazole and on the copolymer of 1-vinyl-1,2,4-triazole with N-vinyl-pyrrolidone was supposed by these authors to be related to low toxicity and high biological activity of these polymeric agents in respect to basidiomycetes *Flammulina velutipes*, *Ganoderma colossus, G. lucidum, G. neojaponicum, Grifola umbellata, Laetiporus sulphureus, Lentinula edodes, Pleurotus ostreatus,* and *Tomophagus cattienensis*. Therefore, the development of vinyltriazole-based materials discussed has provided the technologically convenient way to obtain the appropriate matrices for SeNPs in order to form polymeric composites.

A comprehensive study of characteristics and features of the biologically active nanocomposites of selenium synthesized chemically would be a precious source of information for further implementation of the data obtained in the promising process of developing the medicinal forms and valuable industrial semi-products. Nanoselenium capped with synthetic polymers is a promising nanomaterial for medicine and technique.

#### 4.1.2. Biogenic Polymer Matrices

Organic supports for inorganic nanostructures could be materials that mainly consist of natural polymers [118]. Waste from non-degradable synthetic polymers is becoming an increasingly serious problem. It is of particular interest to develop alternative natural tools. Increasing research focuses on the development of materials with biodegradable properties, renewable polymers from natural sources categorized as biopolymers [119]. Biopolymers are excellent raw materials proven as a good host matrix for various nanostructures. Bio-based polymer films reinforced with nanomaterials have become an interesting area of research [118]. Biocomposites produced from natural materials and degradable inorganic fillers are efficient and sustainable green composite materials [22,120,121]. Biopolymers are capable of exhibiting high efficiency in the stabilization of SeNPs.

Currently, the most investigated polymer for NP incorporation is a polysaccharide chitosan, the only cationic pseudonatural polymer, which is the second most abundant biopolymer in nature [122]. It has excellent properties owing to its antimicrobial activity against Gram-positive and Gram-negative bacteria [123]. Chitosan is extracted from the exoskeleton of arthropods and the cell wall of fungi and may also be obtained synthetically by the deacetylation of chitin [124]. Chitosan is a linear copolymer of β-(1-4)-2-amido-2-deoxy-d-glucan (glucosamine) and β-(1-4)-2-acetamido-2-deoxy-d-glucan (acetylglucosamine) [125]. The biodegradation, biocompatibility, and nontoxic properties of chitosan have been studied extensively [123,126]. Solubility in neutral and basic solutions can be achieved by structural modification, such as carboxylation of the hydroxyl group, which was used to produce carboxymethyl chitosan. The degree of carboxymethylation, which is controlled by the reaction temperature and duration, strongly affects the solubility of carboxymethyl chitosan [127].

Chitosan and carboxymethyl chitosan are appropriate capping and stabilizing agents in a facile synthetic approach to synthesize monodispersible SeNPs. Chen et al. [125] reported on a novel wet chemistry method for the synthesis of chitosan+SeNPs or (carboxymethyl chitosan)+SeNPs nanocomposites by using sodium selenite as a precursor, ascorbic acid as a reducing agent, and potassium iodide as a stabilizer. In this technique, SeNPs were synthesized through a simple oxidation–reduction reactions system also comprising chitosan or carboxymethyl chitosan to form the corresponding nanocomposites afterward.

Frequently used supporting material matrices for NPs are also based on natural compounds and their derivatives, such as alginate, sodium alginate, agar [128], arabinoxylan, xyloglucan [22], glucomannan [129], galactomannan [130,131], hyaluronic acid [132], starch [133,134], pectin [135,136], arabinogalactan [137,138,139,140,141,142,143,144], and carrageenan. Kappa carrageenan (*κ*-carrageenan) is an industrial product, a biocompatible natural sulfated polysaccharide extracted from red algae. The degree of sulfation is about 6%, which corresponds to one sulfate group per disaccharide unit of *κ*-carrageenan macromolecule. It is an appropriate matrix for NPs in polymer nanocomposites [130,131,145]. Water-soluble nanocomposites consisting of SeNPs stabilized by *κ*-carrageenan possess a complex of biological activity [146].

Thus, polysaccharides are good shells for SeNPs, which prevents their clustering and provides this nanomaterial with useful properties. For instance, anti-inflammatory activity of natural polysaccharide-modified SeNPs is proven [147,148]. Features common to polysaccharides as themselves are low toxicity, high biodegradability, biocompatibility, and bioadhesivity [149,150]. Composites based on natural polysaccharides are bioactive, biocompatible, biodegradable, and antibacterial.

**Figure 2:**
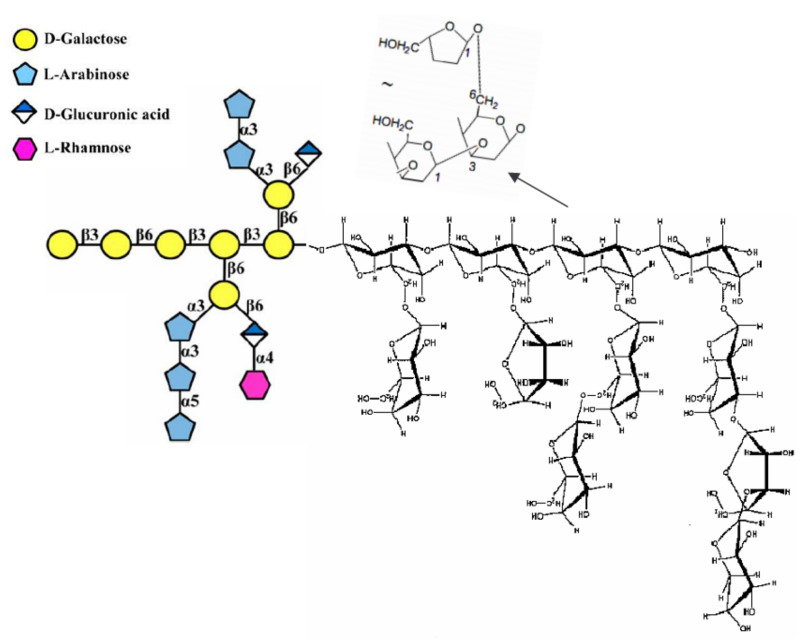
Schematic structure of fragment of arabinogalactan.

In comparison with α-glucans like starch and dextran linked by α-glycosidic bonds, or heteropolysaccharides with both α- and β-glycosidic bonds [151] such as arabinogalactan (Figure 2), β-d-glucans have their glucose units connected by either β-1,3, 1,6, and/or -1,4 glycosidic linkages [152].

**Figure 3:**
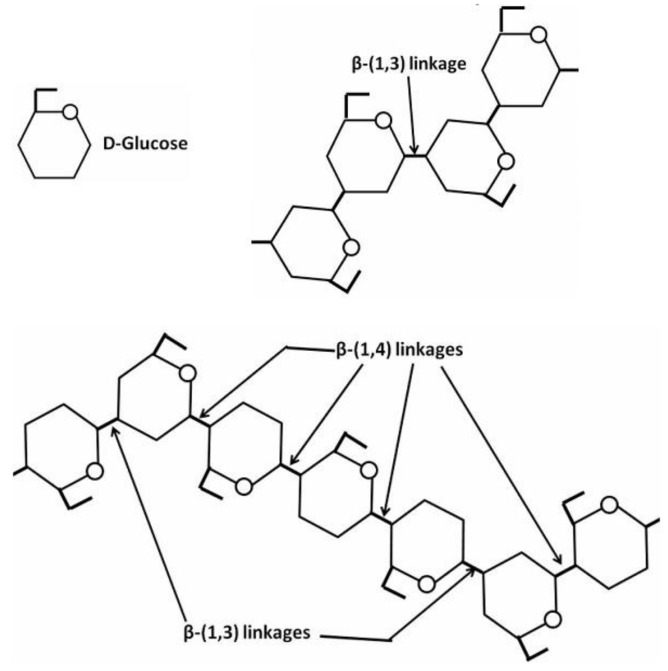
Schematic structure of fragments of β-(1,3)– and β-(1,3),(1,4)–glucans.

A fragment of β-1,3 glucan and an example of glucan with a backbone composed of β-(1,4) linked units interspersed with single β-(1,3) linkages are depicted by Figure 3.

β-d-glucans have exhibited an especially innovative potential, related in large part to their desirable biocompatibility and versatile structural modification via numerous hydroxy functional groups [29] for loading with desirable agents and NPs. Functional peculiarities of β-d-glucans are closely associated with their physicochemical properties such as solubility, conformation (random coils, double or triple helix, rod-like shapes, or spherical structure [153]), and branching characteristics (the position, degree, and length of branching) [154,155,156]. The spherical hyper-branched β-d-glucans could likely be considered a promising carrier for loading with inorganic NPs [157,158].

Mushroom can serve as a rich source of hyper-branched β-d-glucans. Many, if not all, basidiomycetes mushrooms contain biologically active polysaccharides in fruit bodies, cultured mycelium, and cultured broth [52]. Polysaccharides obtained from fungi can display a wide range of valuable physicochemical characteristics, which have driven the interest in research into their functional properties. Fungal polysaccharides possess biological activities with great potential to treat various diseases owing to their immunomodulatory activities [29]. These natural polymers, being advantageous over other natural or synthetic polymers, have found applications in food, pharmaceutical, medical, and cosmetic areas. To address some of the emerging applications, one should mention the development of bionanocomposites based on different fungal polysaccharides, as well as the potential demonstrated by Se-containing ones.

In comparison with other common β-d-glucans applied in nanotechnology, hyper-branched β-d-glucans of fungal origin likely present novel material with emerging research focused on their interaction with SeNPs. Hyper-branched β-d-glucans have a large number of terminal hydroxyl groups, which are numerous reaction sites for possible chemical modification and functionalization, as well as large surface area and shell structure [29], i.e., ideal characteristics for coating SeNPs to form polymeric nanocomposites. For instance, the mushroom sclerotia of *Polyporus rhinocerus* were reported to be characterized by a high proportion (70 mass percent of dry weight) of β-d-glucan [159]. Sclerotia extract prepared with cold alkali and implementing sonication contained a hyper-branched glucan with β-1,3:β-1,6-linked backbone and a branching degree of 0.85. Additionally, hot water extracted from this mushroom’s sclerotia, a polysaccharide–protein complex with a highly branched (degree of branching 0.60) β-d-mannoglucan [160], was also of remarkable bioactivity and showed immunomodulatory effects on bone marrow dendritic cells [161]. Obtained from the fruit bodies of *Ganoderma sinense* mushroom, a water-soluble hyper-branched β-d-glucan had a β-1,6-linked backbone with the side chain composed of β-1,3- and β-1,4-linked D-glucopyranosyl chains [162]. Another novel alkali-soluble hyper-branched β-d-glucan featuring a spherical shape when dispersed in water, and a main chain of β-1,4 linkages branched with β-1,3 and/or β-1,6 linkages, was extracted from the sclerotia of *Pleurotus tuber-regium* mushroom [163]. The creation of highly stable SeNPs was successfully carried out by means of a facile redox system in the presence of this hyperbranched polysaccharide as a stabilizing and capping agent. In a more recent work, SeNPs were decorated with polysaccharides extracted from several mushrooms, as *Polyporus rhinocerus*, *Ganoderma lucidum*, and *Coriolus versicolor* [157]. The above fungal polysaccharide-modified SeNPs could be kept stable for more than two months.

Mushroom cultivation with the prospect of bioactive compounds (various biopolymers, selected low-molecular-mass agents) presents a resourceful biotechnological approach widely used with different basidiomycetes [164,165]. Mushroom metabolites are responsible for the reduction of Se-containing agents to yield selenium nuclei in liquid culture. Mycelial biomass itself and cell-free culture liquid as spent mushroom substrate could serve as remarkable sources of polysaccharides to manufacture polymeric nanocomposites with microelements including selenium. Mycelia formed by growing pure cultures under the submerged conditions are high-quality, consistent, safe, predictable, and economical mushroom products [166,167], and a suitable alternative to yield mushroom product fortified with selenium. At the in vivo assays with mushroom growth on liquid nutrient media, not only polysaccharides, but also other biomolecules present in mushrooms with appropriate bioavailability, biocompatibility, and low toxicity, such as phenolics, terpenoids, etc., could assist fabrication and stabilization of SeNPs. Hydroxyl groups of the extracellular mushroom polysaccharides prevent the SeNP aggregation by intermolecular hydrogen bonds. Thus, nanoselenium at the mushroom’s submerged cultivation is coated by capping agents for particle stabilization and covered by fungal extracellular biopolymers, mainly polysaccharides, in an aqueous environment of mushroom culture. The surface charge of SeNPs is an important determinant of cellular uptake. The NP surface is usually charged in water; thus, one should take into consideration a new energy term associated with electrostatic interactions (e.g., the repulsion energy) [109]. Mushroom polysaccharides are good stabilizers of SeNPs; the latter form a novel complex with exopolysaccharides in submerged culture, with synergistic activity and physicochemical properties favorable for SeNPs’ antibacterial and antibiofilm properties [82,168].

### 4.2. Nanoscale Building Blocks for Selenium Nanocomposites

#### 4.2.1. Selenium Intrinsic Properties Decisive in Nanophase Selection

Nanomaterials such as NPs with their small size and higher specific surface area are appropriate to be immobilized into solid matrices and to construct nanocomposites by modification of NPs with polymer chains. The embedment of NPs into polymeric matrices minimizes their mobility and their interaction with the environment. Thus, the use of nanocomposites in an effective way by increasing their stability, along with the safety of nanoparticles, is of paramount concern. An important advantage of nanocomposites is the accessibility of substrates to the functional NPs [169].

Various inorganic fillers are widely employed to produce polymer composites exhibiting unique physicochemical properties that guarantee active effects, such as antimicrobial effects (e.g., Ag and Cu), scavenging of gas molecules (e.g., Fe or Pd), and antioxidant effects (e.g., selenium) [32], along with the enhanced polymer’s intrinsic properties (thermal, mechanical, optical, rheological properties, etc.) [170]. Thus, along with the metals-containing formulations, the nanoscale fillers in the form of selenium NPs were examined. Moreover, SeNPs constitute in many cases a cheaper and safer alternative because of the selenium role as an essential micronutrient required by living organisms. Selenium has found its path into the area of nanotechnology due to its remarkable biological properties, such as antibacterial, antiviral, and antioxidant characteristics [171].

Selenium biochemistry-related studies are forced to rely on controversial mechanisms built into selenium substances’ chemical nature and intrinsic properties. That gives reasons for the contradictions found among the Se necessary as an essential microelement in human and animal organisms and its toxicity even at moderate levels. An excess amount of selenium causes toxicity and harmful consequences in humans [172]. That is especially true and became commonly accepted about a decade ago with traditional selenium compounds, inorganic salts such as selenite and selenate, which exhibit poor application potential due to the low bioactivity and inadequate cytotoxicity to normal cells [173]. Nanotechnology has revolutionized the field of Se-substances application by expounding SeNPs as a distinctive chemopreventive agent, which shows good selective cellular uptake and enhanced anticancer activities. The acute toxicity of SeNPs in mice is remarkably lower compared to inorganic salts or amino acids, such as selenite, selenomethionine, and selenomethylselenocysteine [174,175]. Along with an anticancer therapy [176,177,178,179], the major biomedical applications of SeNPs include a targeted drug delivery, drug delivery vehicles and artificial enzymes [180,181], and biosensors and intracellular analysis [182]. SeNPs are known for inhibiting cellular entry of viruses while maintaining low toxicity. Several functionalized SeNPs are capable of blocking the pathogen attachment to cell and viral entry, because viral infections start with the binding of viral particles to receptors on the host cells, followed by the entry of the virus into the cells [183]. Selenium-containing NPs, such as zanamivir-functionalized SeNPs [184], SeNPs loaded with oseltamivir [185], amantadine-functionalized SeNPs [186], and SeNPs loaded with ribavirin [187] have been examined for inhibitory effects upon and biocompatibility with the H1N1 influenza virus. The antiviral activity of SeNPs can be further enhanced when being combined with the antiviral drug arbidol [188], effectively blocking cell entry of influenza virus and reducing cell apoptosis. Some drug-conjugated SeNPs were tested against Covid-19 infection and apoptosis inhibition properties [183].

SeNPs can be prepared via physical methodologies such as laser ablation, UV radiation, hydrothermal techniques, etc. [189]. Additionally, SeNPs can be synthesized via chemical methodologies such as acid decomposition, the precipitation method, reduction using ascorbic acid, sodium dodecyl sulfate, sulfur(IV) oxide and glucose, etc. Yet, despite the design of nanoparticles with a definite shape and size via these methods, they require the use of chemicals, harsh conditions like acidic pH, and high temperature, which make them unsuitable for applications in the medical field [2]. As a type of nanomaterial with improved biocompatibility, SeNPs were reported to be obtained via the selenite anions reduction to elemental selenium Se^0^ with polymeric or non-polymeric reducing agents, as chitosan– or another polysaccharide–protein complexes, *Spirulina* polysaccharides, *Undaria pinnatifida* polysaccharide, doxorubicin, or adenosine triphosphate [190,191,192,193,194,195,196]. In the presence of different chemically obtained or pseudonatural polymers serving as a stabilizing agent, SeNPs can be synthesized via the reduction of sodium selenite with, e.g., ascorbic acid [9] by means of a solution-phase approach. Characterization of size and morphology of the resultant NPs by TEM showed that SeNPs obtained using different stabilizers such as chitosan (poly(D-glucosamine), Triton X-100 (t-octylphenoxypolyethoxy ethanol), and ethoxylate-type additive (2,4,7,9-tetramethyl-5-decyne-4,7-diol ethoxylate) were spherical with a diameter within the intervals 20–40, 18–40, and 28–60 nm, respectively. With a stabilizer isotridecanol ethoxylate, SeNPs were of nanorods morphology and exhibited poor antioxidant properties [9]. Subsequently, the solutions containing the selected spherical, 50–60 nm in diameter SeNPs were incorporated in a flexible multilayer plastic material to be used as food packaging. The antioxidant performance and final laminate structure stability assays showed that the Se-layer was a non-migrating, efficient, free-radical scavenger [9].

Therapeutic benefits of SeNPs include anti-inflammatory, anti-diabetic, antioxidant, and antimicrobial action [197]. Many studies indicate potent antitumor activity of SeNPs, their conjugates, and functionalized products [198,199] by inducing caspases and mitochondria-mediated apoptosis [157]. Various conjugates of SeNPs contribute greatly to an emerging interdisciplinary area, cancer nanotechnology. Green synthesis of SeNPs with the experimentally confirmed ability to fight against a wide array of cancer types was performed with degreased walnut meal [200], non-pathogenic bacteria [201,202,203], algae [193], yeast fermented broth [204], and culture extract of fungus *Monascus purpureus* [205]. Mushroom’s *Polyporus rhinocerus* water-soluble polysaccharide–protein complexes [194] and hyper-branched β-d-glucan from *Pleurotus tuber-regium* [157,163] are examples of higher-fungal polymers as the capping agents potentiating in vivo anticancer efficacy of SeNPs. An essential advantage of biogenic SeNPs is to represent more cytotoxicity on cancer cells compared to normal cells. Very recent studies also regard evidence for the anticancer potential of biogenic SeNPs and discuss the proposed anticancer mechanisms of this nanomaterial [206].

The known surface chemistry of the mycogenic SeNPs would lead to the possibility of selecting the reducing molecules and capping agents and thereby control the size and shape of the NPs. In this respect, the chemical form of Se^0^ precursor (nanophase producing) compounds should be considered as a limiting factor determining the subsequent surface modification of neonatal SeNPs in solution. SeNPs show attractive reduced toxicity compared with selenium bulk counterpart (bulk selenium) [207]. Nevertheless, the native SeNPs suffer from the issue of stability, which leads to compromised efficiency and physicochemical characteristics of these nanostructures [208].

#### 4.2.2. Mechanisms Underlying the Occurrence of Selenium Nanophase

Various attempts have been made to improve the stability and biocompatibility of SeNPs. The latter form aggregates, which may have led to less availability and efficacy, resulting in the requirement for a high concentration [209]. To obtain an excellent integration between organic and inorganic phases in nanocomposites, tuning specific procedures should be implemented that allow one to achieve strong polymer–filler interaction. Much effort has been exerted to develop a “green” method of synthesis and dispersion of SeNPs. The generally accepted approach is that the key mechanism behind a green synthesis of NPs is a microorganism- or plant-assisted reduction of precursor compound owing to various metabolites [210], with not one biomolecule but several secondary metabolites together being accountable [211]. Ligand capping agents, otherwise called stabilizing or functionalizing agents, are molecules that bind to the nanoparticle surface to confer stability and prevent nanoparticle aggregation [212]. Unlike physicochemical methods of nanoparticle synthesis, which require additional steps for surface functionalization, in biogenic methods, synthesis and capping occur simultaneously [213]. Likewise, in fungi, biopolymers play a vital role as reducing and capping agents [214]. However, there are few reports on the exact characterization and identification of the capping biomolecules in biogenic NPs. Fungal proteins, enzymes, cofactors, and other metabolites play crucial roles in the organism’s survival and reduce precursor compounds to cause a corresponding chemical element, including selenium, zero-valent nanoparticulate forms.

NPs are assembled from the corresponding atoms, molecules, and clusters using chemical or biological techniques, these constructive procedures being called the bottom-up method in contrast to the top-down (destructive) method of the NPs’ preparation [215]. The bottom-up approach provides definite advantages in terms of better control over the final product formation and homogeneity of physicochemical parameters. Disadvantages inherent to this approach, namely the necessity of separation and purification of the synthesized particles from their reaction mixture, toxic chemicals, organic solvents, and reagents [216], is a serious challenge. The exception is the green synthesis method, in which bottom-up protocol-based procedures of NPs synthesis may be performed under ecofriendly conditions.

NPs can be generated by microbes either intracellularly or extracellularly. Several reviews focus on the bioproduction of metal NPs by fungi [25] and other microorganisms, both unicellular and multicellular [217], to explore the chemistry of inorganic nanomaterial formation through these two methods. In both cases, during the SeNP synthesis, selenium is involved in the nutrient exchange and/or substance diffusion. The microbial cells prevent damage by producing specific metabolites and electrons, which can reduce the Se-containing precursor compound to yield selenium in a higher (zero) oxidation state. The nuclei grow and thereby form nanoparticles intracellularly or extracellularly. Using fungal cells, biosynthesis of SeNPs follows either of the two mechanisms.

In intracellular synthesis, NPs are formed and localized in the cytoplasm, cell wall, or cell membrane. [24]. The fungal cell wall typically contains glucans, glycoproteins, and chitin, which are bonded by inter- and intra-molecular hydrogen-bonding between these units. Glucans contain the predominantly linear β-1,3-linkage and a small portion of β-1,6- and β-1,4-linkages. Chitin and α-1,3-glucan build a hydrophobic scaffold that is surrounded by a hydrated matrix of diversely linked β-glucans. Glycoproteins and a minor fraction of α-1,3-glucans form a highly dynamic shell coating the cell wall surface [218]. Hence, owing to its composition, the fungal cell wall along with various proteins plays a central role in the bioreduction responsible for NPs’ appearance. Alternatively, the NPs or precursor ions may diffuse through the cell membrane and be reduced by redox mediators in the cytoplasmic matrix [219]. Intracellular production of metal NPs by fungi was known for a relatively prolonged period. At the very beginning of the 21st century, the intracellular preparation of AuNPs using a fungus (*Verticillium sp*.) was first reported by Mukherjee et al. [220], where Au^3+^ ions from tetrachloroaurate were reduced within the fungal cells, resulting in the formation of particles within the size range of 20 nm. In the process of intracellular mycogenic synthesis of SeNPs, selenium as a precursor material of NPs is reduced by the fungal biomolecules present in the cell wall to yield NPs, which are formed on the surface of mycelia, not in the nutrient medium. This method presumes that a Se-supplying compound is delivered inside the targeted fungus and creates SeNPs in the company of fungal reducing agents. This nanoparticle precursor (Se-containing compound) first interacts with oppositely charged cell surface moieties, where it could be simultaneously reduced to respective SeNPs and remain bound to the cell surface [32]. Such NPs may diffuse to the cell membrane or cytoplasm. Alternatively, a Se-supplying compound could be internalized by active or passive transport inside the cell, and selenium in zero oxidation state occurs on account of intracellular reducing agents.

Since the intracellularly produced NPs are synthesized by microorganisms including fungi, the purity of particles from this process can be questionable. There is a high possibility that the NPs obtained are associated with the microorganism itself, various microbial cellular components, or both [221,222,223]. Multicomponent residuals from microorganisms accumulated on the NPs surface would also trigger potential immunological reactions when exposed to living systems [16]. Intracellular processes suffer from a significant disadvantage in terms of product recovery that makes the process hard and expensive, since NPs bind to the cell, and certain treatments such as cell disruption or solvent extraction are required to isolate the generated NPs [48]. As SeNPs can also be formed intracellularly, the separation of these particles from the fungal biomass without altering their properties is extremely challenging. The ideal situation would be that SeNPs are produced extracellularly, which turns out to be more environmentally friendly and cost-effective [224].

The extracellular synthesis of NPs was first reported by Shahverdi and co-workers [225], where AgNPs were produced by the reduction of aqueous Ag^+^ ions through various culture supernatants of Gram-negative bacteria. Extracellular mycogenic synthesis of SeNPs involves the bioreduction of the selenium-supplying compound to elemental selenium, which may be stabilized by organic molecules presented in fungal culture liquid, and SeNPs are formed outside the fungal cells. These processes were long ago known to be performed by different biopolymers [226] and other active components including amino acids, vitamins, alkaloids, polyphenols, flavonoids, and organic acids [227], which can act both as reducing and capping agents during the NPs formation, thus promoting the synthesis of NPs and inhibiting their agglomeration [228]. Zhang et al. [229] have demonstrated that the presence of a protein such as bovine serum albumin in the redox system can control the aggregation of elemental selenium atoms, thus inhibiting the formation of bulky red elemental selenium particles and consequently leading to the formation of red elemental selenium nanoparticles. However, the exact events of SeNPs synthesis have not been elucidated yet.

The cellular defense mechanisms manifested by the fungus-mediated NPs’ formation were noted some considerable time ago, mainly during the metal NPs’ mycosynthesis studies [34,35]. It is supposed that fungi will take measures when the toxic ions are present in their growth environment to provide protection [230]. In doing so, fungi secrete extracellular metabolites to be used to remove unwanted Se-containing substance in the form of SeNPs. The exhibited defense mechanisms can be categorized again into intracellular and extracellular means. The former facilitates the reduction of the negative effects of entrapped Se-supplying substance by binding with biomolecules or efflux channels, while the latter implies the inhibition of the uptake and internalization of foreign Se-containing matter [24].

**Figure 4:**
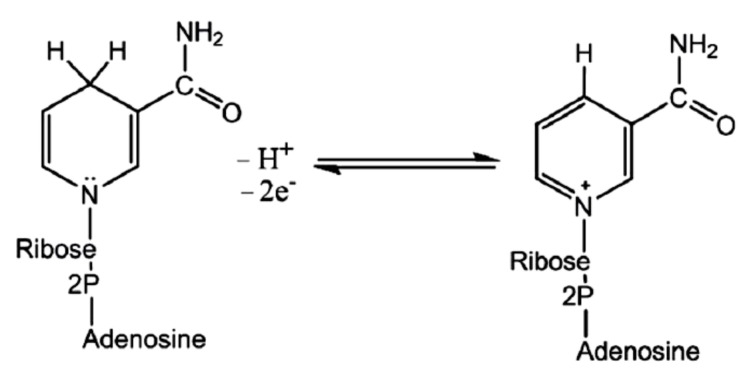
Reversible transitions among NADH and NAD^+^.

Fungal synthesis of NPs takes place with nitrate-dependent reductases and electron shuttle quinones [231]. Fungi produce napthoquinones and anthraquinones [232,233,234,235], which act as reducing agents. Many filamentous fungi elaborate a large number of simple hydroxyl or methoxy derivatives of benzoquinones, toluquinones, or quinoline [219] as a response to abiotic stress. For reduction processes, not only the biocatalyst, enzyme, is necessary, but also an electron shuttle. Membrane-bound oxidoreductases play a crucial role in the process along with different quinones [236]. It was speculated that a conjugation between the quinone electron shuttle with the reductase somehow facilitated the formation of NPs [237]. As early as 2010, Jha and Prasad [236] found that the presence of these metabolites may generate a redox reaction due to tautomerization, leading to the production of nanomaterials. The aforementioned processes transfer electrons to the quinone pool. Additionally, it was expected that fungi secrete a reduced nicotinamide adenine dinucleotide (NADH) as one of the components of the reducing agents moiety [26], which along with other ingredients, reduce the precursor substances to the corresponding NPs. In order to confirm their hypothesis, NADH alone and NADH along with a fungal extract were added to the solution with the precursor agent (to be reduced to yield NPs). The researchers did not observe any change in color for NADH alone. However, when NADH accompanied by a fungal extract was added, the reaction started after a few minutes [26]. The assays show that NADH could be a key factor in the synthesis of NPs, but other molecules are essential, perhaps, as a biocatalyst of the redox reaction [238]. The reductase enzyme obtains its electrons from NADH oxidation to an oxidized nicotinamide adenine dinucleotide (NAD^+^) (Figure 4).

During the oxidation, the enzyme also becomes oxidized simultaneously, resulting in NPs’ synthesis. Since NADH acts as an electron carrier and selenium in Se-supplying precursor as an electron acceptor, the reduction of positively charged Se species to SeNPs occurs. It has been observed that the nitrate-dependent reductase can also participate in the bioreduction [215]. Some studies reported that NADH-dependent reductases could be specific to a fungal species [42,239], e.g., reductase specific to *Fusarium oxysporum* enzyme was believed to catalyze the facilitated NPs formation [240,241]. Thus, the above-mentioned extracellular defense mechanism behind the SeNPs mycosynthesis is believed to involve NADH-dependent enzymes, notably nitrate reductases, which are secreted into the reaction medium together with electron shuttles such as quinones.

Smaller-molecular-mass substances such as ascorbate, citrate [242], cofactors, glucose, and amino acids [243] could also serve as reducing agents, which stabilize the resulting NPs in fungal biosystems. Some studies dealing with the fungi-assisted fabrication of SeNPs suggest electrostatic interaction of free amine groups or cysteine residues with NPs’ surface [244,245,246,247]. The electrostatic interaction followed by secretion of such metabolites as extracellular polymeric substances that can adhere to unwanted foreign matters (NP material) [248] are the first steps of the fungal SeNPs’ formation mechanism. Stimulation of fungal development by Se-containing molecules is owing to the fungus capability of binding and stabilizing key compounds for mycelium growth, whose molecules have various chemical structures, dimensional and charge characteristics, solubility, lipophilic properties, and reactivity. The distribution of charges plays a certain role. The negative charge on the mycelium surface, which promotes the complexation process during selenium–ligand interactions, is provided by chitin, a component of the fungal cell wall [249], and carboxyl, amine, thiol, amide, imine, thioether, and phosphate functional groups [250]. That is why the electrostatic interaction is believed to happen between the positively charged Se-sourcing matter (inorganic Se-salts or organic selenide DAPS-25) and the negatively charged groups on the fungal cell. While the electrophilic character of Se(IV) and Se(VI) is of no doubt, the electrophilicity of Se(II) in the molecule of selenide (Figure 3) is confirmed by the positive value of the natural charge on the selenium atom, as was computed by Pankratov A.N. [247].

**Figure 5:**
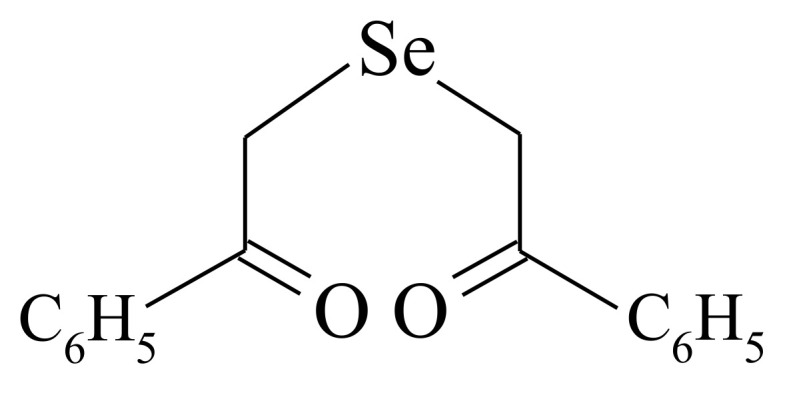
Structure of the compound DAPS-25.

**Figure 6:**
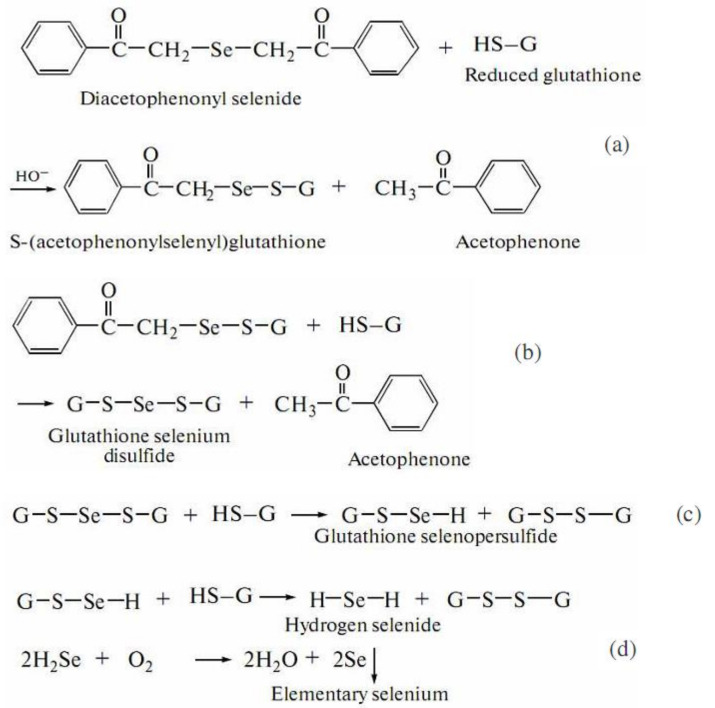
Scheme of DAPS-25 interaction with thiols.

Molecular mechanism studies suggested that certain NPs inactivate proteins by binding with -SH (thiol) groups of proteins in the cell. Tripeptide L-γ-glutamyl-γ-cysteinyl-glycine, glutathione (GSH), contains an active thiol group of a cysteine residue. GSH secretion is a very important element of redox homeostasis [251]. Due to a sufficient negative value of redox potential, GSH acts as a buffering agent in the eukaryotic cells [19]. It has been found that GSH secretion under abiotic stress initiates the intracellular detoxification pathway. Several schemes are known for the reaction of organoselenium compounds with thiols. In the work [246], aimed at the investigation of a reaction between DAPS-25 (Figure 5) and cysteine or reduced glutathione for their identification by the ascending thin-layer chromatography technique, a scheme for the interaction of diacetophenonyl selenide with the compound-bearing sulfhydryl group was proposed (Figure 6).

The half-products, acetophenone and S-(acetophenylselenyl)glutathione, form in the first step (Figure 6a). The next step is the formation of one acetophenone molecule and glutathione selenodisulfide (Figure 6b). Then, glutathione selenopersulfide and glutathione disulfide form from selenodisulfide in an excess of reduced glutathione (Figure 6c). The final step is the formation of hydrogen selenide. The resulting hydrogen selenide as a strong reductant can be oxidized to elementary selenium by both air oxygen and glutathione disulfide (Figure 6d) [246]. Therefore, for an organoselenium compound to be decomposed rapidly under the action of reduced glutathione or cysteine to release elementary selenium, its molecule should contain the –CO–CH_2_–Se–CH_2_–CO– group.

**Figure 7:**
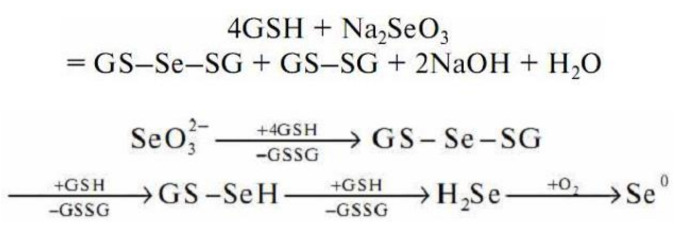
Scheme of sodium selenite interaction with reduced glutathione.

Sodium selenite undergoes a non-enzymatic reaction with GSH to form selenodiglutathione (GS–Se–SG) [252] (Figure 7).

According to the general scheme of the redox reaction, in an excess of GSH, selenodiglutathione is readily reduced to form selenopersulfide (GSSeH) and then hydrogen selenide. Hydrogen selenide oxidizes to elemental selenium.

Thus, there are fungal protective mechanisms to resist the toxicity of the Se-precursor compounds by their immobilization in a less-toxic Se^0^ nanoparticulate form. The eukaryotic microbial system as fungus has enough strategies to survive with selenite stress. Exposure to the Se-sourcing agent solution prompts the fungus to produce metabolites to overcome xenobiotic-induced stress. In this process, the Se-supplying substances are reduced to SeNPs through the catalytic effect of the extracellular enzymes and by implementing the fungal metabolites. The defense option applied by fungi to survive in the presence of unwanted Se-agent consists in the binding of Se-ions to high-affinity functional groups, with thiols, such as cysteine residues and GSH, contributing greatly to this fungal strategy. An elemental selenium accumulation presents the final stage (Figure 6 and Figure 7).

Various organic and inorganic chemical compounds may serve as a reducing agent implemented in mechanisms of SeNPs mycosynthesis. Along with the low-molecular-weight compounds considered above, mushroom biopolymers, mainly bioactive polysaccharides, could serve as the capping agent during the reduction of selenium salts [194] and organoselenium substances. Fungal polysaccharides could engage the surface of SeNPs to prevent particulate aggregation and control their particle size [157]. Strong physical adsorption of hydroxyl groups on selenium surfaces aids in the development and stabilization to construct water-dispersible SeNPs capped with a polymeric agent in an aqueous system [163]. However, the precise processes are unclear, and further research should provide a deeper understanding of the molecular mechanisms involved in the occurrence of selenium nanophase.

#### 4.2.3. Points for Physicochemical Characterization of Culture Conditions and Resultant SeNPs

Before being implemented in any branch of technology, the nanomaterial needs to be fully understood and characterized [253]. The physicochemical characterization of NPs complements their biochemical characterization. Many articles describe techniques that are effective in the characterization of nanostructures and nanomaterials. Particle attributes such as composition, size distribution, morphology, surface chemistry, homogeneity, stability, etc., are crucial for determining the biotechnological potentialities and the impact of the NP-comprising matter on the environment. Reliable analytical tools employed for SeNP characterization yield multidimensional information on these nanostructures. The anthropogenic and naturally occurring SeNPs are usually characterized via several techniques such as ultraviolet-visible (UV-Vis) absorbance spectroscopy, light-scattering-based techniques (dynamic light scattering (DLS) and nanoparticles tracking analysis (NTA)) [153], Fourier transform infrared (FTIR) spectroscopy [254], transmission electron microscopy (TEM) and field emission scanning electron microscopy (FESEM) [9], X-ray diffraction (XRD) measurements, and Raman spectroscopic techniques [255]. Qualitative analysis for selenium is frequently carried out with electron energy loss spectroscopy in the TEM (EELS-TEM) accompanied by a chromatographic (gas chromatography coupled with mass spectrometry, GC-MS) detection of the Se-supplying compound (bio)transformation in the course of the biosynthetic process [81]. Hyphenated techniques are efficiently applied for selenium speciation, e.g., inductively coupled plasma mass spectrometry (ICP-MS) [256] in single-particle mode (SP-ICP-MS) or coupled with asymmetric flow field-flow fractionation (AF^4^) in AF^4^-ICP-MS [257], and continuous photochemical vapor generation (PCVG) coupled with microwave-induced plasma optical emission spectrometry (MIP-OES). Bartosiak et al. applied the yeasts *Saccharomyces boulardii* to SeNPs bioproduction and performed calculation of accurate NP yield [258] A selective identification and quantitative determination of both the unreacted precursor Se(IV) and the resultant NPs without the need to separate them were enabled by means of the PCVG- MIP-OES technique.

External physicochemical parameters of the Se^0^ producing fermentation, such as acidity, temperature, aeration, Se-supplying compound type and concentration, etc., are found to be important factors that could be decisive for the SeNPs nanoscale features. Well-dispersed spherical 60 nm sized SeNPs synthesized at pH 8 had an average diameter value of 300 nm in strongly acidic conditions [259]. Acidity parameters close to those of neutral solutions (pH 7 to 8) [260] or a somewhat wider pH range (pH 6 to 9 for precursor selenite and pH 7 to 9 for selenate salt) [261] were reported to facilitate the SeNPs synthesis. However, for some microorganisms used in green techniques, far-from-neutral media can be appropriate. The corresponding pH range was as broad as 4 to 10 when bacterium *Acinetobacter* sp. SW30 was implemented for SeNPs bioproduction [201]. In this research, the SeNP formation process occurred under thermal conditions up to 40 °C, and much higher temperature (80 to 100 °C) appeared to cause the SeNPs’ aggregation into nanorods. The effect of broad acidity parameter (pH 5 to 12) on the expression of proteins in the fungus *Mariannaea* sp. HJ in the presence of selenium oxide SeO_2_ as a Se-precursor was demonstrated [262]. It was indicated that the higher the acidity, the greater the total protein concentration, and both maxima were reached at pH 10–11 and 198 mg/L, respectively. Further increase in pH value led to a drastic decrease in the total protein concentration. Thus, pH could affect the expression of fungal proteins capable of alleviating alkaline condition stress in the fungus. The Se-sourcing agent’s concentration along with the cultivation pattern of biosynthesizing microorganism manages the size, shape, and cellular location of SeNP [255]. Spherical and rod-like SeNP shapes were produced at 3.0 mM of sodium selenite in the medium, whereas Se-spheres were solely found at 1.5 mM of the same starting Se-compound [201]. Aeration is one of the influencing factors here. Diko et al. reported the synthesis of spherical SeNPs (≤200 nm in diameter) using *Pseudomonas stutzeri*, which reduced sodium selenate more rapidly under anaerobic conditions, while Se(IV) salt was not reduced at all. Strain NT-I was able to tolerate elevated concentrations of inorganic Se-salts. Bacterium reduced selenate completely at its level of up to 10 mM and selenite almost completely (up to 9 mM salt concentration). In addition, even higher concentrations of these Se-salts were substantially reduced by the bacterium [263]. Moderate thermal (20 to 50 °C) and acidity (pH 7–9 or 6–9 for selenite and selenate, respectively) conditions were favorable for bioreduction to yield elemental selenium.

Since common physicochemical factors control the cellular location of SeNPs, both the intracellular and extracellular locations of mycosynthesized SeNPs were observed by many researchers [33,262,264]. Thus, filamentous fungi *Aureobasidium pullulans*, *Mortierella humilis*, *Trichoderma harzianum,* and *Phoma glomerata* were capable of intra- and extracellular bioproduction of SeNPs [33]. The red deposit was confirmed as elemental selenium during fungal growth on Se-containing media. Along with Se^0^ formation, selenium oxide was found in the case of *Trichoderma harzianum* culture with 1 mM selenite. Both particle sizes and SeNPs concentrations were determined by the SP-ICP-MS technique. Only *A. pullulans* and *M. humilis* produced SeNPs in 10-day culture, with diameters ca. 60 and 48 nm, respectively. In 20- and 30-day cultures, particles were enlarged in diameter to reach about 78 and 61 nm. Using the SP-ICP-MS method, only a low SeNP level was detected in *T. harzianum* and *P. glomerata* supernatants of liquid cultures [33]. Rosenfeld et al. showed that the ascomycete fungi (*Pyrenochaeta* sp., *Acremonium strictum*, *Plectosphaerella cucumerina*, *Stagonospora* sp., *Alternaria alternata*, *Paraconiothyrium sporulosum*) produced Se^0^ by Se inorganic salts reduction, with the NPs being strongly bound by the fungal biomass for all six species used [264]. SeNPs’ diameter ranged from 50–100 nm (*Alternaria alternata*) to 200–300 nm (other fungal species). Particles were spherical. Results of XRD analysis demonstrated that the diffraction patterns of all SeNPs were X-ray amorphous, although two of the analyzed extracellular SeNPs mycosynthesized by *Acremonium strictum* were crystalline with a d-spacing of 0.37 nm. Residual dissolved Se(IV) and Se(VI) concentration values were determined by conductivity measurements using an ion chromatography system enabling a simultaneous quantification of both oxyanions, comprising Se(IV) and Se(VI), with a detection limit of micromole/L for each anion. Analysis for total selenium associated with fungal biomass was performed using the inductively coupled plasma optical emission spectroscopy (ICP-OES). The presence of Se^0^ was confirmed by SEM EDS. Transmission electron microscopy revealed intracellular and extracellular SeNPs in all fungal species used [264]. Biosynthesis of SeNPs by *Mariannaea* sp. HJ was reported for the first time very recently [262]. Various culture conditions were studied, including SeO_2_ concentrations and pH of reaction media. To characterize the resultant SeNPs, the multimethod approach was used: UV-Vis, TEM, SEM, XRD, FTIR, and sodium dodecyl sulfate-polyacrylamide gel electrophoresis (SDS-PAGE) were used. TEM images depicted the SeNPs’ deposition both on the fungal cell wall and in the cytoplasmic region, thus suggesting the biotransformation of Se(IV) into elemental selenium to occur in extracellular and intacellular locations, which could provide a template for these bioreduction processes [262].

## 5. Conclusions and Challenges

The production of NPs and polymer nanocomposites is the area of nanotechnology that is growing exponentially. Nanomaterials can be synthesized by several chemical and physical approaches, and owing to the recent green-oriented research, it is possible to integrate the use of biological entities. Microorganisms could be deemed as cell factories for nanosized bioactive materials fabrication. Microorganism-assisted nanoparticle biosynthesis should be regarded as a viable green option. The contemporary scientific literature addressing the element-containing polymer nanocomposites testifies to the considerable attention given to selenium-nanoparticles comprising ones.

The presence of the polymeric matrix that immobilizes SeNPs is extremely important, somehow tuning the nanophase properties to compromise for a real-life application of selenium nanocomposites. Manufacturing of Se-containing polymer nanocomposites using fungi is attractive, since biomolecules like fungal polymers and low-molecular-mass compounds secreted by the fungal biomass can serve as reducing and capping agents during the nanosynthesis process. Higher fungi, mushrooms, are edible and medicinal eukaryotic microorganisms that are cost-effective and able to produce SeNPs and Se-containing polymeric biocomposites. The strategy of utilization of fungal metabolites for subsequent formation of SeNPs provides the new exciting possibility of selenium nanocomposite bioproduction and assists the development of am ecofriendly fungal biopolymer-based large-scale bioprocess for several biomedical, food, and agrochemical applications due to these bioproducts’ potent properties.

Interpretation of the mechanisms of SeNPs and polymeric Se-nanocomposites production using fungi is still in its infancy. Future-focused research is needed to more clearly and deeply elucidate the exact mechanisms of the Se-supplying precursor reduction reactions to yield selenium in a zero oxidation state, SeNPs stabilizing reactions followed by the interaction with capping agents, incorporation into suitable biodegradable matrices as possible hosts for Se-nanomodifiers, etc.

An open challenge is the development of processes to fabricate Se-nanomaterials with controlled and tunable properties, and environmentally friendly alternatives include mycosynthesized SeNPs and Se-nanocomposites based on fungal biopolymers. However, very few published works are intended to supply the readers with conclusive information on the biomolecules involved in Se-source reduction leading to the SeNPs’ occurrence and the capping agents of fungal origin responsible for the stability of Se-nanocomposites. Mushrooms’ ability to synthesize SeNPs extracellularly should be treated as undoubtedly advantageous in this regard. One of the most significant contributing factors to the nano-biosynthetic mechanism seems to be the choice of suitable species and strains of fungi used in parallel with the optimization of diversified conditions required for fungal culture. With ongoing efforts in improving nanomaterials’ synthesis efficiency and meanwhile exploring the environmental risks associated with those, it is hopeful that the myconanotechnology potentialities move to a fundamentally new level and their commercial applications in biotechnological sectors will be realized in the coming years.

## Data Availability

The data presented in this study is available in this article.

## References

[1] Gade A., Ingle A., Whiteley C., Rai M. (2010). Mycogenic metal nanoparticles: Progress and applications. Biotechnol. Lett..

[2] Wadhwani S.A., Shedbalkar U.U., Singh R., Chopade B.A. (2016). Biogenic selenium nanoparticles: Current status and future prospects. Appl. Microbiol. Biotechnol..

[3] Castro L., Blázquez M.L., Muñoz J.A., González F.G., Ballester A. (2014). Mechanism and Applications of Metal Nanoparticles Prepared by Bio-Mediated Process. Rev. Adv. Sci. Eng..

[4] Pathakoti K., Manubolu M., Hwang H.-M. (2017). Nanostructures: Current uses and future applications in food science. J. Food Drug Anal..

[5] Wilson P., Ke P.C., Davis T.P., Kempe K. (2017). Poly(2-oxazoline)-based micro- and nanoparticles: A review. Eur. Polym. J..

[6] Singla R., Abidi S.M.S., Dar A., Acharya A. (2019). Nanomaterials as potential and versatile platform for next generation tissue engineering applications. J. Biomed. Mater. Res. Part B Appl. Biomater..

[7] Oguz O., Simsek E., Soz C.K., Heinz O.K., Yilgor E., Yilgor I., Menceloglu Y.Z. (2018). Effect of filler content on the structure-property behavior of poly(ethylene oxide) based polyurethaneurea-silica nanocomposites. Polym. Eng. Sci..

[8] Gatadi S., Madhavi Y.V., Nanduri S. (2021). Nanoparticle drug conjugates treating microbial and viral infections: A mini-review. J. Mol. Struct..

[9] Vera P., Echegoyen Y., Canellas E., Nerín C., Palomo M., Madrid Y., Cámara C. (2016). Nano selenium as antioxidant agent in a multilayer food packaging material. Anal. Bioanal. Chem..

[10] Divya K., Kurian L.C., Vijayan S., Manakulam Shaikmoideen J. (2016). Green synthesis of silver nanoparticles by Escherichia coli: Analysis of antibacterial activity. J. Water Environ. Nanotechnol..

[11] Huang Z.-H., Yin Y.-N., Zhang Y. (2016). Preparation of a novel positively charged nanofiltration composite membrane incorpo-rated with silver nanoparticles for pharmaceuticals and personal care product rejection and antibacterial properties. Water Sci. Technol..

[12] Talib S.H., Challab M.K., Alhameedawi F.A.H. (2021). Using Nano Composites to Purify Water from Phenol Pollutants. J. Phys. Conf. Ser..

[13] Colino C.I., Lanao J.M., Gutierrez-Millan C. (2021). Recent advances in functionalized nanomaterials for the diagnosis and treatment of bacterial infections. Mater. Sci. Eng. C.

[14] Martínez G., Merinero M., Pérez-Aranda M., Pérez-Soriano E., Ortiz T., Villamor E., Begines B., Alcudia A. (2020). Environmental Impact of Nanoparticles’ Application as an Emerging Technology: A Review. Materials.

[15] Wang E., Wang A.Z. (2014). Nanoparticles and their applications in cell and molecular biology. Integr. Biol..

[16] Rónavári A., Igaz N., Adamecz D., Szerencsés B., Molnar C., Kónya Z., Pfeiffer I., Kiricsi M. (2021). Green Silver and Gold Nanoparticles: Biological Synthesis Approaches and Potentials for Biomedical Applications. Molecules.

[17] Salunke B.K., Sawant S.S., Lee S.-I., Kim B.S. (2016). Microorganisms as efficient biosystem for the synthesis of metal nanoparticles: Current scenario and future possibilities. World J. Microbiol. Biotechnol..

[18] Wang Q., Wang F., Xu Z., Ding Z. (2017). Bioactive Mushroom Polysaccharides: A Review on Monosaccharide Composition, Biosynthesis and Regulation. Molecules.

[19] Geetha N., Bhavya G., Abhijith P., Shekhar R., Dayananda K., Jogaiah S. (2021). Insights into nanomycoremediation: Secretomics and mycogenic biopolymer nanocomposites for heavy metal detoxification. J. Hazard. Mater..

[20] Oréfice R.L., Hench L.L., Brennan A.B. (2001). Effect of particle morphology on the mechanical and thermo-mechanical behavior of polymer composites. J. Braz. Soc. Mech. Sci..

[21] Morgan A.B., Chu L.L., Harris J.D. (2005). A flammability performance comparison between synthetic and natural clays in poly-styrene nanocomposites. Fire Mater Int. J..

[22] Khan M.A., Razak S.A., Al Arjan W., Nazir S., Anand T.S., Mehboob H., Amin R. (2021). Recent Advances in Biopolymeric Composite Materials for Tissue Engineering and Regenerative Medicines: A Review. Molecules.

[23] Zan G., Wu Q. (2016). Biomimetic and Bioinspired Synthesis of Nanomaterials/Nanostructures. Adv. Mater..

[24] Priyadarshini E., Priyadarshini S.S., Cousins B.G., Pradhan N. (2021). Metal-Fungus interaction: Review on cellular processes underlying heavy metal detoxification and synthesis of metal nanoparticles. Chemosphere.

[25] Hulkoti N.I., Taranath T.C. (2014). Biosynthesis of nanoparticles using microbes—A review. Colloids Surf. B Biointerfaces.

[26] Li G., He D., Qian Y., Guan B., Gao S., Cui Y., Yokoyama K., Wang L. (2012). Fungus-mediated green synthesis of silver na-noparticles using Aspergillus terreus. Int. J. Mol. Sci..

[27] Pantidos N., Horsfall L.E. (2014). Biological synthesis of metallic nanoparticles by bacteria, fungi and plants. J. Nanomed. Nanotechnol..

[28] Taherzadeh M.J., Fox M., Hjorth H., Edebo L. (2003). Production of mycelium biomass and ethanol from paper pulp sulfite liquor by Rhizopus oryzae. Bioresour. Technol..

[29] Su Y., Chen L., Yang F., Cheung P.C.K. (2021). Beta-d-glucan-based drug delivery system and its potential application in tar-geting tumor associated macrophages. Carbohydr. Polym..

[30] Bhavya G., Belorkar S.A., Mythili R., Geetha N., Shetty H.S., Udikeri S.S., Jogaiah S. (2021). Remediation of emerging environ-mental pollutants: A review based on advances in the uses of eco-friendly biofabricated nanomaterials. Chemosphere.

[31] Poluboyarinov P.A., Vikhreva V.A., Leshchenko P.P., Aripovskii A.V., Likhachev A.N. (2009). Elemental selenium formation upon destruction of the organoselenium compound DAFS-25 molecule by growing fungal mycelium. Mosc. Univ. Biol. Sci. Bull..

[32] Afzal B., Yasin D., Husain S., Zaki A., Srivastava P., Kumar R., Fatma T. (2019). Screening of cyanobacterial strains for the selenium nanoparticles synthesis and their anti-oxidant activity. Biocatal. Agric. Biotechnol..

[33] Liang X., Perez M.A.M.-J., Nwoko K.C., Egbers P., Feldmann J., Csetenyi L., Gadd G.M. (2019). Fungal formation of selenium and tellurium nanoparticles. Appl. Microbiol. Biotechnol..

[34] Mukherjee P., Ahmad A., Mandal D., Senapati S., Sainkar S.R., Khan M.I., Parishcha R., Ajaykumar P.V., Alam M., Kumar R. (2001). Fungus-Mediated Synthesis of Silver Nanoparticles and Their Immobilization in the Mycelial Matrix: A Novel Biological Approach to Nanoparticle Synthesis. Nano Lett..

[35] Priyabrata M., Ahmad A., Deendayal M., Satyajyoti S., Sudhakar R.S., Mohammad I.K., Renu P., Ajaykumar P.V., Mansoor A., Rajiv K. (2001). Fungus mediated synthesis of silver nanoparticles and their immobilization in the mycelial matrix: A novel biological approach to nanoparticle synthesis. Nano Lett..

[36] Estevez M.B., Raffaelli S., Mitchell S.G., Faccio R., Alborés S. (2020). Biofilm Eradication Using Biogenic Silver Nanoparticles. Molecules.

[37] Crisan C.M., Mocan T., Manolea M., Lasca L.I., Tăbăran F.-A., Mocan L. (2021). Review on Silver Nanoparticles as a Novel Class of Antibacterial Solutions. Appl. Sci..

[38] Sharma G., Nam J.S., Sharma A.R., Lee S.S. (2018). Antimicrobial potential of silver nanoparticles synthesized using medicinal herb coptidis rhizome. Molecules.

[39] Vamanu E., Ene M., Bita B., Ionescu C., Craciun L., Sarbu I. (2018). In Vitro Human Microbiota Response to Exposure to Silver Nanoparticles Biosynthesized with Mushroom Extract. Nutrients.

[40] Kitching M., Ramani M., Marsili E. (2015). Fungal biosynthesis of gold nanoparticles: Mechanism and scale up. Microb. Biotechnol..

[41] Zhang S., Pang G., Chen C., Qin J., Yu H., Liu Y., Zhang X., Song Z., Zhao J., Wang F. (2019). Effective cancer immunotherapy by Ganoderma lucidum polysaccharide-gold nanocomposites through dendritic cell activation and memory T cell response. Carbohydr. Polym..

[42] Rai M., Bonde S., Golinska P., Trzcińska-Wencel J., Gade A., Abd-Elsalam K., Shende S., Gaikwad S., Ingle A. (2021). *Fusarium* as a Novel Fungus for the Synthesis of Nanoparticles: Mechanism and Applications. J. Fungi.

[43] Siddiqi K.S., Husen A. (2016). Fabrication of metal nanoparticles from fungi and metal salts: Scope and application. Nanoscale Res. Lett..

[44] Khandel P., Shahi S.K. (2018). Mycogenic nanoparticles and their bio-prospective applications: Current status and future challenges. J. Nanostruct. Chem..

[45] Khan A.U., Malik N., Khan M., Cho M.H., Khan M.M. (2018). Fungi-assisted silver nanoparticle synthesis and their applications. Bioprocess Biosyst. Eng..

[46] Salunke B.K., Sawant S.S., Lee S.I., Kim B.S. (2015). Comparative study of MnO2 nanoparticle synthesis by marine bacterium Sac-charophagus degradans and yeast Saccharomyces cerevisiae. Appl. Microbiol. Biotechnol..

[47] Athira K., Gurrala L., Kumar D.V.R. (2021). Biosurfactant-mediated biosynthesis of CuO nanoparticles and their antimicrobial activity. Appl. Nanosci..

[48] Zare B., Babaie S., Setayesh N., Shahverdi A.R. (2013). Isolation and characterization of a fungus for extracellular synthesis of small selenium nanoparticles. Nanomedicine.

[49] Mosallam F.M., Gharieb S., EleSayyad G.S., Fathy R.M., EleBatal A.I. (2018). Biomoleculesemediated synthesis of selenium na-noparticles using Aspergillus oryzae fermented Lupin extract and gamma radiation for hindering the growth of some mul-tidrugeresistant bacteria and pathogenic fungi. Microb. Pathog..

[50] Agnihotri M., Joshi S., Kumar A.R., Zinjarde S., Kulkarni S. (2009). Biosynthesis of gold nanoparticles by the tropical marine yeast Yarrowia lipolytica NCIM 3589. Mater. Lett..

[51] Haq M., Rathod V., Singh D., Singh A.K., Ninganagouda S., Hiremath J. (2015). Dried mushroom Agaricus bisporus mediated synthesis of silver nanoparticles from Bandipora District (Jammu and Kashmir) and their efficacy against methicillin resistant Staphylococcus aureus (MRSA) strains. Nanosci. Nanotechnol. Int. J..

[52] Chang S.-T., Wasser S.P. (2012). The Role of Culinary-Medicinal Mushrooms on Human Welfare with a Pyramid Model for Human Health. Int. J. Med. Mushrooms.

[53] Chang S.T., Miles P.G. (1992). Mushroom biology—A new discipline. Mycologist.

[54] Lindequist U., Niedermeyer T.H.J., Jülich W.-D. (2005). The Pharmacological Potential of Mushrooms. Evid.-Based Complement. Altern. Med..

[55] Sharifi-Rad J., Butnariu M., Ezzat S.M., Adetunji C.O., Imran M., Sobhani S.R., Tufail T., Hosseinabadi T., Ramírez-Alarcón K., Martorell M. (2020). Mushrooms-Rich Preparations on Wound Healing: From Nutritional to Medicinal Attributes. Front. Pharmacol..

[56] Vamanu E., Pelinescu D. (2017). Effects of mushroom consumption on the microbiota of different target groups–Impact of poly-phenolic composition and mitigation on the microbiome fingerprint. LWT-Food Sci. Technol..

[57] Vamanu E., Nita S. (2014). Biological activity of fluidized bed ethanol extracts from several edible mushrooms. Food Sci. Biotechnol..

[58] Vamanu E. (2017). Bioactive capacity of some Romanian wild edible mushrooms consumed mainly by local communities. Nat. Prod. Res..

[59] Gergely V., Kubachka K.M., Mounicou S., Fodor P., Caruso J.A. (2006). Selenium speciation in Agaricus bisporus and Lentinula edodes mushroom proteins using multi-dimensional chromatography coupled to inductively coupled plasma mass spectrometry. J. Chromatogr. A.

[60] Falandysz J. (2008). Selenium in Edible Mushrooms. J. Environ. Sci. Health Part C.

[61] Klimaszewska M., Górska S., Dawidowski M., Podsadni P., Turło J. (2016). Biosynthesis of Se-methyl-seleno-L-cysteine in Ba-sidiomycetes fungus Lentinula edodes (Berk.) Pegler. SpringerPlus.

[62] Maseko T., Callahan D.L., Dunshea F., Doronila A., Kolev S., Ng K. (2013). Chemical characterisation and speciation of organic selenium in cultivated selenium-enriched Agaricus bisporus. Food Chem..

[63] Turło J., Gutkowska B., Malinowska E. (2007). Relationship between the selenium, selenomethionine, and selenocysteine content of submerged cultivated mycelium of Lentinula edodes (Berk.). Acta Chromatogr..

[64] Egressy-Molnár O., Ouerdane L., Győrfi J., Dernovics M. (2016). Analogy in selenium enrichment and selenium speciation between selenized yeast Saccharomyces cerevisiae and Hericium erinaceous (lion’s mane mushroom). LWT.

[65] Turło J., Gutkowska B., Herold F., Gajzlerska W., Dawidowski M., Dorociak A., Zobel A. (2011). Biological Availability and Preliminary Selenium Speciation in Selenium-Enriched Mycelium ofLentinula edodes (Berk.). Food Biotechnol..

[66] Espinosa-Ortiz E.J., Gonzalez-Gil G., Saikaly P.E., Van Hullebusch E.D., Lens P.N.L. (2014). Effects of selenium oxyanions on the white-rot fungus Phanerochaete chrysosporium. Appl. Microbiol. Biotechnol..

[67] Niedzielski P., Mleczek M., Siwulski M., Gąsecka M., Kozak L., Rissmann I., Mikołajczak P. (2014). Efficacy of supplementation of selected medicinal mushrooms with inorganic selenium salts. J. Environ. Sci. Health Part B.

[68] Milovanović I.N. (2014). Ability of Selenium Absorption and Biological Activity of Mycelial Extracts of Selected Basidiomycotina Species. Ph.D. Thesis.

[69] Turło J., Gutkowska B., Herold F. (2010). Effect of selenium enrichment on antioxidant activities and chemical composition of Lentinula edodes (Berk.) Pegl. mycelial extracts. Food Chem. Toxicol..

[70] Gharieb M.M., Wilkinson S.C., Gadd G.M. (1995). Reduction of selenium oxyanions by unicellular, polymorphic and filamentous fungi: Cellular location of reduced selenium and implications for tolerance. J. Ind. Microbiol. Biotechnol..

[71] Milovanović I., Brčeski I., Stajić M., Korać A., Vukojević J., Knežević A. (2014). Potential of Pleurotus ostreatus Mycelium for Se-lenium Absorption. Sci. World J..

[72] Goyal A., Kalia A., Sodhi H.S. (2015). Selenium stress in Ganoderma lucidum: A scanning electron microscopy appraisal. Afr. J. Microbiol. Res..

[73] Muñoz A.H.S., Kubachka K., Wrobel K., Corona J.F.G., Yathavakilla S.K.V., Caruso J.A., Wrobel K. (2006). Se-Enriched Mycelia ofPleurotus ostreatus: Distribution of Selenium in Cell Walls and Cell Membranes/Cytosol. J. Agric. Food Chem..

[74] Milovanovic I., Brceski I., Stajic M., Knezevic A., Vukojevic J. (2013). Potential Enrichment of Medicinal Mushrooms with Selenium to Obtain New Dietary Supplements. Int. J. Med. Mushrooms.

[75] Thiry C., Ruttens A., De Temmerman L., Schneider Y.-J., Pussemier L. (2012). Current knowledge in species-related bioavailability of selenium in food. Food Chem..

[76] Vinceti M., Maraldi T., Bergomi M., Malagoli C. (2009). Risk of Chronic Low-Dose Selenium Overexposure in Humans: Insights From Epidemiology and Biochemistry. Rev. Environ. Health.

[77] Hazane-Puch F., Champelovier P., Arnaud J., Garrel C., Ballester B., Faure P., Laporte F. (2013). Long-term selenium supple-mentation in HaCaT cells: Importance of chemical form for antagonist (protective versus toxic) activities. Biol. Trace Elem. Res..

[78] Drevko B.I., Drevko R.I., Antipov V.A., Chernukha B.A., Yakovlev A.N. (2001). Remedy for Treatment and Prophylactics of In-fectious Diseases and Poisonings of Animals and Poultry Enhancing Their Productivity and Vitality (in Russian). Russian Federation Patent.

[79] Pankratov A.N., Loshchinina E.A., Tsivileva O.M., Burashnikova M.M., Kazarinov I.A., Bylinkina N.N., Nikitina V.E. (2012). Effects of xenobiotic organoselenium compound on the growth and metabolism of basidiomycete Lentinula edodes culture. Izv. Saratov Univ. New Series. Ser. Chem. Biol. Ecol..

[80] Tsivileva O.M., Loshchinina E.A., Pankratov A.N., Burashnikova M., Yurasov N.A., Bylinkina N.N., Kazarinov I.A., Nikitina V.E. (2012). Biodegradation of an Organoselenium Compound to Elemental Selenium by Lentinula edodes (Shiitake) Mushroom. Biol. Trace Element Res..

[81] Drevko Y.B., Sitnikova T.S., Burov A.M., Drevko B.I., Shchegolev S.Y. (2016). Reduction of diacetophenonyl selenide (DAPS-25 formulation) to acetophenone with the formation of selenium micro-and nanoparticles in the presence of Saccharomyces cerevisiae culture. Appl. Biochem. Microbiol..

[82] Tsivileva O.M., Perfileva A.I. (2017). Selenium compounds biotransformed by mushrooms: Not only dietary sources, but also toxicity mediators. Curr. Nutr. Food Sci..

[83] Behera M., Ram S. (2014). Inquiring the mechanism of formation, encapsulation, and stabilization of gold nanoparticles by poly(vinyl pyrrolidone) molecules in 1-butanol. Appl. Nanosci..

[84] Ahmadi Y., Ahmad S. (2020). Recent progress in the synthesis and property enhancement of waterborne polyurethane nanocom-posites: Promising and versatile macromolecules for advanced applications. Polym. Rev..

[85] Tao A.R., Habas S., Yang P. (2008). Shape Control of Colloidal Metal Nanocrystals. Small.

[86] Fayaz A.M., Balaji K., Girilal M., Yadav R., Kalaichelvan P.T., Venketesan R. (2010). Biogenic synthesis of silver nanoparticles and their synergistic effect with antibiotics: A study against gram-positive and gram-negative bacteria. Nanomed. Nanotechnol. Biol. Med..

[87] Aktürk A., Erol Taygun M., Karbancıoglu Güler F., Goller G., Küçükbayrak S. (2019). Fabrication of antibacterial polyvinylalcohol nanocomposite mats with soluble starch coated silver nanoparticles. Colloids Surf. A Physicochem. Eng. Asp..

[88] Wei X., Cai J., Lin S., Li F., Tian F. (2021). Controlled release of monodisperse silver nanoparticles via in situ cross-linked polyvinyl alcohol as benign and antibacterial electrospun nanofibers. Colloids Surf. B Biointerfaces.

[89] Siddiqui N., Bhardwaj A., Hada R., Yadav V.S., Goyal D. (2018). Synthesis, characterization and antimicrobial study of poly (methyl methacrylate)/Ag nanocomposites. Vacuum.

[90] Philip P., Jose T., Parameswaran M., Thankaraj S. (2021). Structurally modified poly(methyl methacrylate) electrospun nanofibers as better host matrix for noble metal nanoparticles. J. Appl. Polym. Sci..

[91] Jatoi A.W. (2020). Polyurethane nanofibers incorporated with ZnAg composite nanoparticles for antibacterial wound dressing applications. Compos. Commun..

[92] Kasi G., Viswanathan K., Sadeghi K., Seo J. (2019). Optical, thermal, and structural properties of polyurethane in Mg-doped zinc oxide nanoparticles for antibacterial activity. Prog. Org. Coatings.

[93] Jafari A., Hassanajili S., Karimi M.B., Emami A., Ghaffari F., Azarpira N. (2018). Effect of organic/inorganic nanoparticles on performance of polyurethane nanocomposites for potential wound dressing applications. J. Mech. Behav. Biomed. Mater..

[94] Alippilakkotte S., Kumar S., Sreejith L. (2017). Fabrication of PLA/Ag nanofibers by green synthesis method using Momordica charantia fruit extract for wound dressing applications. Colloids Surf. A Physicochem. Eng. Asp..

[95] Zhang H.Y., Jiang H.B., Kim J.-E., Zhang S., Kim K.-M., Kwon J.-S. (2020). Bioresorbable magnesium-reinforced PLA membrane for guided bone/tissue regeneration. J. Mech. Behav. Biomed. Mater..

[96] Sportelli M.C., Picca R.A., Cioffi N. (2016). Recent advances in the synthesis and characterization of nano-antimicrobials. TrAC Trends Anal. Chem..

[97] Ditaranto N., van der Werf I.D., Picca R.A., Sportelli M.C., Giannossa L.C., Bonerba E., Tantillo G., Sabbatini L. (2015). Charac-terization and behaviour of ZnO-based nanocomposites designed for the control of biodeterioration of patrimonial stone-works. New J. Chem..

[98] Zare E.N., Makvandi P., Borzacchiello A., Tay F.R., Ashtari K., Padil V.V.T. (2019). Antimicrobial gum bio-based nanocomposites and their industrial and biomedical applications. Chem. Commun..

[99] Stevanović M., Filipović N., Djurdjević J., Lukić M., Milenković M., Boccaccini A. (2015). 45S5Bioglass®-based scaffolds coated with selenium nanoparticles or with poly (lactide-co-glycolide)/selenium particles: Processing, evaluation and antibacterial ac-tivity. Colloids Surf. B Biointerfaces.

[100] Ermakova T.G., Kuznetsova N.P., Pozdnyakov A.S., Larina L., Korzhova S.A., Mazyar I.V., Shcherbakova V.S., Ivanov A.V., Mikhaleva A.I., Prozorova G.F. (2016). 1-Vinyl-1,2,4-triazole in copolymerization reaction with 1-vinyl-4,5,6,7-tetrahydroindole: Synthesis and properties of copolymers. Russ. Chem. Bull..

[101] Fernández-Ortuño D., Torés J.A., de Vicente A., Pérez-García A. (2008). Mechanisms of resistance to QoI fungicides in phyto-pathogenic fungi. Int. Microbiol..

[102] Shcherbakova L.A. (2019). Fungicide resistance of plant pathogenic fungi and their chemosensitization as a tool to increase an-ti-disease effects of triazoles and strobilurines. Sel’skokhozyaistvennaya Biol. [Agric. Biol. ].

[103] Pobezhimova T.P., Korsukova A.V., Dorofeev N., Grabelnych O.I., Siberian Institute of Plant Physiology and Biochemistry SB RAS (2019). Physiological effects of triazole fungicides in plants. Proc. Univ. Appl. Chem. Biotechnol..

[104] Yin B., Ma H., Wang S., Chen S. (2003). Electrochemical Synthesis of Silver Nanoparticles under Protection of Poly(N-vinylpyrrolidone). J. Phys. Chem. B.

[105] Kvitek L., Panáček A., Soukupova J., Kolar M., Vecerova R., Prucek R., Holecová M., Zboril R. (2008). Effect of surfactants and polymers on stability and antibacterial activity of silver nanoparticles (NPs). J. Phys. Chem. C.

[106] Sintubin L., De Windt W., Dick J., Mast J., Van Der Ha D., Verstraete W., Boon N. (2009). Lactic acid bacteria as reducing and capping agent for the fast and efficient production of silver nanoparticles. Appl. Microbiol. Biotechnol..

[107] Bakshi P.S., Selvakumar D., Kadirvelu K., Kumar N.S. (2020). Chitosan as an environment friendly biomaterial–a review on recent modifications and applications. Int. J. Biol. Macromol..

[108] Chen S.-F., Zhang H. (2012). Aggregation kinetics of nanosilver in diferent water conditions. Adv. Nat. Sci. Nanosci. Nanotechnol..

[109] Zhang C., Hu Z., Deng B. (2016). Silver nanoparticles in aquatic environments: Physiochemical behavior and antimicrobial mechanisms. Water Res..

[110] Alexandridis P. (2010). Gold Nanoparticle Synthesis, Morphology Control, and Stabilization Facilitated by Functional Polymers. Chem. Eng. Technol..

[111] Ermakova T.G., Shaulina L.P., Kuznetsova N.P., Prozorova G.F. (2017). Synthesis and sorption activity of copolymers of vinyl-triazole with diethylene glycol divinyl ether. Russ. Chem. Bull..

[112] Ermakova T.G., Shaulina L.P., Kuznetsova N.P., Ratovskii G.V., Soboleva I.N., Pozdnyakov A.S., Prozorova G.F. (2012). Sorption recovery of noble metal ions with a copolymer of 1-vinyl-1,2,4-triazole with acrylonitrile. Russ. J. Appl. Chem..

[113] Ermakova T.G., Shaulina L.P., Kuznetsova N.P., Volkova L.I., Pozdnyakov A.S., Prozorova G.F. (2012). Sorption of noble metal compounds by cross-linked copolymer of 1-vinyl-1,2,4-triazole with acrylic acid. Russ. J. Appl. Chem..

[114] Aslan A., Bozkurt A. (2009). Development and characterization of polymer electrolyte membranes based on ionical cross-linked poly(1-vinyl-1,2,4 triazole) and poly(vinylphosphonic acid). J. Power Sources.

[115] Dzhardimalieva G.I., Uflyand I.E. (2018). Synthetic Methodologies for Chelating Polymer Ligands: Recent Advances and Future Development. ChemistrySelect.

[116] Tikhonov N.I., Khutsishvili S.S., Larina L., Pozdnyakov A.S., Emel’Yanov A.I., Prozorova G.F., Vashchenko A.V., Vakul’Skaya T.I. (2019). Silver polymer complexes as precursors of nanocomposites based on polymers of 1-vinyl-1,2,4-triazole. J. Mol. Struct..

[117] Tsivileva O.M., Perfileva A.I., Ivanova A.A., Pozdnyakov A.S., Prozorova G.F. (2021). The Effect of Selenium- or Met-al-Nanoparticles Incorporated Nanocomposites of Vinyl Triazole Based Polymers on Fungal Growth and Bactericidal Properties. J. Polym. Environ..

[118] Jamróz E., Kulawik P., Kopel P. (2019). The Effect of Nanofillers on the Functional Properties of Biopolymer-Based Films: A Review. Polymers.

[119] John M.J., Thomas S. (2008). Biofibres and biocomposites. Carbohydr. Polym..

[120] Trache D., Hussin M.H., Chuin C.T.H., Sabar S., Fazita M.N., Taiwo O.F., Hassan T., Haafiz M.M. (2016). Microcrystalline cellulose: Isolation, characterization and bio-composites application—A review. Int. J. Biol. Macromol..

[121] Khan M.U.A., Haider S., Haider A., Kadir M.R.A., Abd Razak S.I., Shah S.A., Javad A., Shakir I., Al-Zahrani A.A. (2020). Devel-opment of Porous, Antibacterial and Biocompatible GO/n-HAp/Bacterial Cellulose/β-Glucan Biocomposite Scaffold for Bone Tissue Engineering. Arab. J. Chem..

[122] Frank L.A., Onzi G.R., Morawski A.S., Pohlmann A.R., Guterres S.S., Contri R.V. (2020). Chitosan as a coating material for na-noparticles intended for biomedical applications. React. Funct. Polym..

[123] Amine R., Tarek C., Hassane E., Noureddine E.H., Khadija O. (2021). Chemical Proprieties of Biopolymers (Chitin/Chitosan) and their Synergic Effects with Endophytic *Bacillus* Species: Unlimited Applications in Agriculture. Molecules.

[124] Baxter A., Dillon M., Taylor K.A., Roberts G.A. (1992). Improved method for i.r. determination of the degree of N-acetylation of chitosan. Int. J. Biol. Macromol..

[125] Chen W., Li Y., Yang S., Yue L., Jiang Q., Xia W. (2015). Synthesis and antioxidant properties of chitosan and carboxymethyl chitosan-stabilized selenium nanoparticles. Carbohydr. Polym..

[126] Kumar M.N.V.R., Muzzarelli R.A.A., Muzzarelli C., Sashiwa H., Domb A.J. (2004). Chitosan chemistry and pharmaceutical per-spectives. Chem. Rev..

[127] Chen X.-G., Park H.-J. (2003). Chemical characteristics of O-carboxymethyl chitosans related to the preparation conditions. Carbohydr. Polym..

[128] Jaiswal L., Shankar S., Rhim J.-W., Hahm D.-H. (2020). Lignin-mediated green synthesis of AgNPs in carrageenan matrix for wound dressing applications. Int. J. Biol. Macromol..

[129] Jiang Y., Huang J., Wu X., Ren Y., Li Z., Ren J. (2020). Controlled release of silver ions from AgNPs using a hydrogel based on konjac glucomannan and chitosan for infected wounds. Int. J. Biol. Macromol..

[130] Lesnichaya M.V., Aleksandrova G.P., Feoktistova L.P., Sapozhnikov A.N., Fadeeva T.V., Sukhov B.G., Trofimov B.A. (2010). Silver-containing nanocomposites based on galactomannan and carrageenan: Synthesis, structure, and antimicrobial properties. Russ. Chem. Bull..

[131] Lesnichaya M.V., Aleksandrova G.P., Sukhov B., Rokhin A.V. (2013). Molecular-weight characteristics of galactomannan and carrageenan. Chem. Nat. Compd..

[132] Rao K.M., Suneetha M., Zo S., Duck K.H., Han S.S. (2019). One-pot synthesis of ZnO nanobelt-like structures in hyaluronan hy-drogels for wound dressing applications. Carbohydr. Polym..

[133] Perfileva A.I., Nozhkina O.A., Graskova I.A., Sidorov A.V., Lesnichaya M.V., Aleksandrova G.P., Dolmaa G., Klimenkov I.V., Sukhov B. (2018). Synthesis of selenium and silver nanobiocomposites and their influence on phytopathogenic bacterium Clavibacter michiganensis subsp. sepedonicus. Russ. Chem. Bull..

[134] Lv H., Cui S., Yang Q., Song X., Wang D., Hu J., Zhou Y., Liu Y. (2021). AgNPs-incorporated nanofiber mats: Relationship between AgNPs size/content, silver release, cytotoxicity, and antibacterial activity. Mater. Sci. Eng. C.

[135] Soubhagya A., Moorthi A., Prabaharan M. (2020). Preparation and characterization of chitosan/pectin/ZnO porous films for wound healing. Int. J. Biol. Macromol..

[136] Hileuskaya K., Ladutska A., Kulikouskaya V., Kraskouski A., Novik G., Kozerozhets I., Kozlovskiy A., Agabekov V. (2020). ‘Green’ approach for obtaining stable pectin-capped silver nanoparticles: Physico-chemical characterization and antibacterial activity. Colloids Surf. A Physicochem. Eng. Asp..

[137] Trofimov B.A., Sukhov B.G., Aleksandrova G.P., Medvedeva S.A., Grishchenko L.A., Mal’Kina A.G., Feoktistova L.P., Sapozhnikov A.N., Dubrovina V.I., Martynovich E. (2003). Nanocomposites with Magnetic, Optical, Catalytic, and Biologically Active Properties Based on Arabinogalactan. Dokl. Chem..

[138] Grishchenko L.A., Medvedeva S.A., Aleksandrova G.P., Feoktistova L.P., Sapozhnikov A.N., Sukhov B.G., Trofimov B.A. (2006). Redox reactions of arabinogalactan with silver ions and formation of nanocomposites. Russ. J. Gen. Chem..

[139] Graskova I.A., Zhivet’Yev M.A., Borovskii G.B., Sukhov B.G. (2012). Bactericide impact of polymer-stabilized multi-functional nano-composites. J. Stress Physiol. Biochem..

[140] Papkina A.V., Perfileva A.I., Zhivetev M.A., Borovskiy G.B., Graskova I.A., Lesnichaya M.V., Klimenkov I.V., Sukhov B.G., Trofimov B.A. (2015). Effect of selenium and arabinogalactan nanocomposite on viability of the phytopathogen Clavibacter michi-ganensis subsp. sepedonicus. Dokl. Biol. Sci..

[141] Papkina A.V., Perfileva A.I., Zhivet’yev M.A., Borovskii G.B., Graskova I.A., Klimenkov I.V., Lesnichaya M.V., Sukhov B.G., Trofimov B.A. (2015). Complex effects of selenium-arabinogalactan nanocomposite on both phytopathogen Clavibacter michi-ganensis subsp. sepedonicus and potato plants. Nanotechn. Russ..

[142] Kolesnikova L., Karpova E.A., Vlasov B.Y., Sukhov B.G., Mov B.A.T. (2015). Lipid Peroxidation–Antioxidant Defense System during Toxic Liver Damage and Its Correction with a Composite Substance Containing Selenium and Arabinogalactan. Bull. Exp. Biol. Med..

[143] Shurygina I.A., Rodionova L.V., Shurygin M., Sukhov B., Kuznetsov S.V., Popova L.G., Dremina N.N. (2015). Using confocal microscopy to study the effect of an original pro-enzyme Se/arabinogalactan nanocomposite on tissue regeneration in a skeletal system. Bull. Russ. Acad. Sci. Phys..

[144] Rodionova L.V., Shurygina I.A., Sukhov B.G., Popova L.G., Shurygin M., Artem’Ev A.V., Pogodaeva N.N., Kuznetsov S.V., Gusarova N.K., Trofimov B. (2015). Nanobiocomposite based on selenium and arabinogalactan: Synthesis, structure, and application. Russ. J. Gen. Chem..

[145] Lesnichaya M.V., Sukhov B., Aleksandrova G.P., Gasilova E.R., Vakul’Skaya T.I., Khutsishvili S.S., Sapozhnikov A.N., Klimenkov I.V., Trofimov B. (2017). Chiroplasmonic magnetic gold nanocomposites produced by one-step aqueous method using κ-carrageenan. Carbohydr. Polym..

[146] Lesnichaya M.V., Shendrik R., Sukhov B.G. (2019). Relation between excitation dependent luminescence and particle size distri-butions for the selenium nanoparticles in κ-carrageenan shell. J. Lumin..

[147] Zhu C., Zhang S., Song C., Zhang Y., Ling Q., Hoffmann P.R., Li J., Chen T., Zheng W., Huang Z. (2017). Selenium nanoparticles decorated with Ulva lactuca polysaccharide potentially attenuate colitis by inhibiting NF-κB mediated hyper inflammation. J. Nanobiotechnol..

[148] Wang J., Zhang Y., Yuan Y., Yue T. (2014). Immunomodulatory of selenium nano-particles decorated by sulfated Ganoderma lucidum polysaccharides. Food Chem. Toxicol..

[149] Perinelli D.R., Fagioli L., Campana R., Lam J.K., Baffone W., Palmieri G.F., Casettari L., Bonacucina G. (2018). Chitosan-based nanosystems and their exploited antimicrobial activity. Eur. J. Pharm. Sci..

[150] Tamer T.M., Hassan M.A., Omer A.M., Valachová K., Eldin M.M., Collins M.N., Šoltés L. (2017). Antibacterial and antioxidative activity of O-amine functionalized chitosan. Carbohydr. Polym..

[151] Liang Y., Faik A., Kieliszewski M., Tan L., Xu W.L., Showalter A.M. (2010). Identification and characterization of in vitro galacto-syltransferase activities involved in arabinogalactan-protein glycosylation in tobacco and Arabidopsis. Plant Physiol..

[152] Kagimura F.Y., da Cunha M.A.A., Barbosa A.M., Dekker R.F., Malfatti C.R.M. (2015). Biological activities of derivatized d-glucans: A review. Int. J. Biol. Macromol..

[153] Kaur R., Sharma M., Ji D., Xu M., Agyei D. (2019). Structural Features, Modification, and Functionalities of Beta-Glucan. Fibers.

[154] Chen L., Ge M.-D., Zhu Y.-J., Song Y., Cheung P.C., Zhang B.-B., Liu L.-M. (2019). Structure, bioactivity and applications of natural hyperbranched polysaccharides. Carbohydr. Polym..

[155] Legentil L., Paris F., Ballet C., Trouvelot S., Daire X., Vetvicka V., Ferrières V. (2015). Molecular interactions of β-(1→ 3)-glucans with their receptors. Molecules.

[156] Tao Y., Zhang L. (2006). Determination of molecular size and shape of hyperbranched polysaccharide in solution. Biopolymers.

[157] Zeng J., Wang R., Zhang S., Fang J., Liu S., Sun G., Xu B., Xiao Y., Fu D., Zhang W. (2019). Hydro-gen-bonding-assisted exogenous nucleophilic reagent effect for β-selective glycosylation of rare 3-amino sugars. J. Am. Chem. Soc..

[158] Li X., Cheung P.C.K. (2019). Application of natural β-d-glucans as biocompatible functional nanomaterials. Food Sci. Hum. Wellness.

[159] Lau B.F., Abdullah N., Aminudin N. (2013). Chemical Composition of the Tiger’s Milk Mushroom, Lignosus rhinocerotis (Cooke) Ryvarden, from Different Developmental Stages. J. Agric. Food Chem..

[160] Liu C., Choi M.W., Li X., Cheung P.C. (2018). Immunomodulatory effect of structurally-characterized mushroom sclerotial polysaccharides isolated from Polyporus rhinocerus on human monoctyes THP-1. J. Funct. Foods.

[161] Liu C., Choi M.W., Xue X., Cheung P.C. (2019). Immunomodulatory effect of structurally characterized mushroom sclerotial polysaccharides isolated from Polyporus rhinocerus on bone marrow dendritic cells. J. Agric. Food Chem..

[162] Han X.-Q., Chan B.C.L., Dong C.-X., Yang Y.-H., Ko C.H., Yue G.G.-L., Chen D., Wong C.-K., Lau C., Tu P.-F. (2012). Isolation, Structure Characterization, and Immunomodulating Activity of a Hyperbranched Polysaccharide from the Fruiting Bodies of Ganoderma sinense. J. Agric. Food Chem..

[163] Zhang Y., Wang J., Zhang L. (2010). Creation of Highly Stable Selenium Nanoparticles Capped with Hyperbranched Polysaccharide in Water. Langmuir.

[164] Chen W., Zhao Z., Li Y. (2011). Simultaneous increase of mycelial biomass and intracellular polysaccharide from Fomes fomentarius and its biological function of gastric cancer intervention. Carbohydr. Polym..

[165] Ruthes A.C., Smiderle F., Iacomini M. (2015). d-Glucans from edible mushrooms: A review on the extraction, purification and chemical characterization approaches. Carbohydr. Polym..

[166] Wasser S.P. (2011). Current findings, future trends, and unsolved problems in studies of medicinal mushrooms. Appl. Microbiol. Biotechnol..

[167] Corrêa R.C.G., Brugnari T., Bracht A., Peralta R.M., Ferreira I.C. (2016). Biotechnological, nutritional and therapeutic uses of Pleurotus spp. (Oyster mushroom) related with its chemical composition: A review on the past decade findings. Trends Food Sci. Technol..

[168] Perfileva A.I., Tsivileva O.M., Koftin O.V., Anis’Kov A.A., Ibragimova D.N. (2018). Selenium-Containing Nanobiocomposites of Fungal Origin Reduce the Viability and Biofilm Formation of the Bacterial Phytopathogen Clavibacter michiganensis subsp. sepedonicus. Nanotechnol. Russ..

[169] Kim J., Van der Bruggen B. (2010). The use of nanoparticles in polymeric and ceramic membrane structures: Review of manufacturing procedures and performance improvement for water treatment. Environ. Poll..

[170] Videira-Quintela D., Martin O., Montalvo G. (2021). Recent advances in polymer-metallic composites for food packaging applica-tions. Trends Food Sci. Technol..

[171] Shakibaie M., Forootanfar H., Golkari Y., Mohammadi-Khorsand T., Shakibaie M.R. (2015). Anti-biofilm activity of biogenic selenium nanoparticles and selenium dioxide against clinical isolates of Staphylococcus aureus, Pseudomonas aeruginosa, and Proteus mirabilis. J. Trace Elements Med. Biol..

[172] Tran P.A., Webster T.J. (2013). Antimicrobial selenium nanoparticle coatings on polymeric medical devices. Nanotechnology.

[173] Zhang Q., Chen L., Guo K., Zheng L., Liu B., Yu W., Guo C., Liu Z., Chen Y., Tang Z. (2013). Effects of Different Selenium Levels on Gene Expression of a Subset of Selenoproteins and Antioxidative Capacity in Mice. Biol. Trace Element Res..

[174] Mennini N., Furlanetto S., Cirri M., Mura P. (2012). Quality by design approach for developing chitosan-Ca-alginate microspheres for colon delivery of celecoxib-hydroxy-propyl-b-cyclodextrin-PVP complex. Eur. J. Pharm. Biopharm..

[175] Khiralla G.M., El-Deeb B.A. (2015). Antimicrobial and antibiofilm effects of selenium nanoparticles on some foodborne pathogens. LWT.

[176] Xu H., Cao W., Zhang X. (2013). Selenium-Containing Polymers: Promising Biomaterials for Controlled Release and Enzyme Mimics. Accounts Chem. Res..

[177] Xia Y., You P., Xu F., Liu J., Xing F. (2015). Novel Functionalized Selenium Nanoparticles for Enhanced Anti-Hepatocarcinoma Activity In vitro. Nanoscale Res. Lett..

[178] Zheng W., Cao C., Liu Y., Yu Q., Zheng C., Sun D., Ren X., Liu J. (2015). Multifunctional polyamidoamine-modified selenium nanoparticles dual-delivering siRNA and cisplatin to A549/DDP cells for reversal multidrug resistance. Acta Biomater..

[179] Poluboyarinov P.A., Elistratov D.G., Moiseeva I.J. (2020). Antitumor Activity of Selenium and Search Parameters for Its New Po-tentially Active Derivatives. Russ. J. Bioorgan. Chem..

[180] Liu T., Zeng L., Jiang W., Fu Y., Zheng W., Chen T. (2015). Rational design of cancer-targeted selenium nanoparticles to antagonize multidrug resistance in cancer cells. Nanomed. Nanotechnol. Biol. Med..

[181] Sun D., Liu Y., Yu Q., Qin X., Yang L., Zhou Y., Chen L., Liu J. (2014). Inhibition of tumor growth and vasculature and fluorescence imaging using functionalized ruthenium-thiol protected selenium nanoparticles. Biomaterials.

[182] Shivaramakrishnan B., Gurumurthy B., Balasubramanian A. (2017). Potential biomedical applications of metallic nanobiomaterials: A review. Int. J. Pharm. Sci. Res..

[183] Medhi R., Srinoi P., Ngo N., Tran H.-V., Lee T.R. (2020). Nanoparticle-Based Strategies to Combat COVID-19. ACS Appl. Nano Mater..

[184] Lin Z., Li Y., Guo M., Xiao M., Wang C., Zhao M., Xu T., Xia Y., Zhu B. (2017). Inhibition of H1N1 influenza virus by selenium nanoparticles loaded with zanamivir through p38 and JNK signaling pathways. RSC Adv..

[185] Li Y., Lin Z., Guo M., Xia Y., Zhao M., Wang C., Xu T., Chen T., Zhu B. (2017). Inhibitory activity of selenium nanoparticles functionalized with oseltamivir on H1N1 influenza virus. Int. J. Nanomed..

[186] Li Y., Lin Z., Guo M., Zhao M., Xia Y., Wang C., Xu T., Zhu B. (2018). Inhibition of H1N1 influenza virus-induced apoptosis by functionalized selenium nanoparticles with amantadine through ROS-mediated AKT signaling pathways. Int. J. Nanomed..

[187] Lin Z., Li Y., Gong G., Xia Y., Wang C., Chen Y., Hua L., Zhong J., Tang Y., Liu X. (2018). Restriction of H1N1 influenza virus infection by selenium nanoparticles loaded with ribavirin via resisting caspase-3 apoptotic pathway. Int. J. Nanomed..

[188] Li Y., Lin Z., Gong G., Guo M., Xu T., Wang C., Zhao M., Xia Y., Tang Y., Zhong J. (2019). Inhibition of H1N1 influenza virus-induced apoptosis by selenium nanoparticles functionalized with arbidol through ROS-mediated signaling pathways. J. Mater. Chem. B.

[189] Iranifam M., Fathinia M., Rad T.S., Hanifehpour Y., Khataee A., Joo S. (2013). A novel selenium nanoparticles-enhanced chemiluminescence system for determination of dinitrobutylphenol. Talanta.

[190] Chen T., Wong Y.-S., Zheng W., Bai Y., Huang L. (2008). Selenium nanoparticles fabricated in Undaria pinnatifida polysaccharide solutions induce mitochondria-mediated apoptosis in A375 human melanoma cells. Colloids Surf. B Biointerfaces.

[191] Huang Y., He L., Liu W., Fan C., Zheng W., Wong Y.-S., Chen T. (2013). Selective cellular uptake and induction of apoptosis of cancer-targeted selenium nanoparticles. Biomaterials.

[192] Wu H., Li X., Liu W., Chen T., Li Y., Zheng W., Man C.W.-Y., Wong M.-K., Wong K.-H. (2012). Surface decoration of selenium nanoparticles by mushroom polysaccharides–protein complexes to achieve enhanced cellular uptake and antiproliferative activity. J. Mater. Chem..

[193] Yang Y., Mathieu J.M., Chattopadhyay S., Miller J.T., Wu T., Shibata T., Guo W., Alvarez P.J. (2012). Defense mechanisms of Pseudomonas aeruginosa PAO1 against quantum dots and their released heavy metals. ACS Nano.

[194] Wu H., Zhu H., Li X., Liu Z., Zheng W., Chen T., Yu B., Wong K.-H. (2013). Induction of Apoptosis and Cell Cycle Arrest in A549 Human Lung Adenocarcinoma Cells by Surface-Capping Selenium Nanoparticles: An Effect Enhanced by Polysaccharide–Protein Complexes from Polyporus rhinocerus. J. Agric. Food Chem..

[195] Yu B., Zhang Y., Zheng W., Fan C., Chen T. (2012). Positive Surface Charge Enhances Selective Cellular Uptake and Anticancer Efficacy of Selenium Nanoparticles. Inorg. Chem..

[196] Zhang Y., Li X., Huang Z., Zheng W., Fan C., Chen T. (2013). Enhancement of cell permeabilization apoptosis-inducing activity of selenium nanoparticles by ATP surface decoration. Nanomed. Nanotechnol. Biol. Med..

[197] Khurana A., Tekula S., Saifi M.A., Venkatesh P., Godugu C. (2019). Therapeutic applications of selenium nanoparticles. Biomed. Pharmacother..

[198] Ramamurthy C., Sampath K.S., Arunkumar P., Kumar M.S., Sujatha V., Premkumar K., Thirunavukkarasu C. (2013). Green synthesis and characterization of selenium nanoparticles and its augmented cytotoxicity with doxorubicin on cancer cells. Bioprocess Biosyst. Eng..

[199] Kumar S., Tomar M.S., Acharya A. (2015). Carboxylic group-induced synthesis and characterization of selenium nanoparticles and its anti-tumor potential on Dalton’s lymphoma cells. Colloids Surf. B Biointerfaces.

[200] Ren J., Liao W., Zhang R., Dong C., Yu Z. (2016). Novel walnut peptide–selenium hybrids with enhanced anticancer synergism: Facile synthesis and mechanistic investigation of anticancer activity. Int. J. Nanomed..

[201] Wadhwani S.A., Gorain M., Banerjee P., Shedbalkar U.U., Singh R., Kundu G.C., Chopade B.A. (2017). Green synthesis of selenium nanoparticles using *Acinetobacter* sp. SW30: Optimization, characterization and its anticancer activity in breast cancer cells. Int. J. Nanomed..

[202] Xu C., Qiao L., Guo Y., Ma L., Cheng Y. (2018). Preparation, characteristics and antioxidant activity of polysaccharides and pro-teins-capped selenium nanoparticles synthesized by Lactobacillus casei ATCC 393. Carbohydr. Polym..

[203] Tabibi M., Agaei S.S., Amoozegar M.A., Nazari R., Zolfaghari M.R. (2020). Antibacterial, Antioxidant, and Anticancer Activities of Biosynthesized Selenium Nanoparticles Using Two Indigenous Halophilic Bacteria. Arch. Hyg. Sci..

[204] Goud K.G., Veldurthi N.K., Vithal M., Reddy G. (2016). Characterization and evaluation of biological and photocatalytic activities of selenium nanoparticles synthesized using yeast fermented broth. J. Mater. Nanosci..

[205] El-Sayed E.S.R., Abdelhakim H.K., Zakaria Z. (2020). Extracellular biosynthesis of cobalt ferrite nanoparticles by Monascus pur-pureus and their antioxidant, anticancer and antimicrobial activities: Yield enhancement by gamma irradiation. Mater. Sci. Eng. C.

[206] Vahidi H., Barabadi H., Saravanan M. (2020). Emerging Selenium Nanoparticles to Combat Cancer: A Systematic Review. J. Clust. Sci..

[207] Huang T.-S., Shyu Y.-C., Chen H.-Y., Lin L.-M., Lo C.-Y., Yuan S.-S., Chen P.-J. (2013). Effect of Parenteral Selenium Supplementation in Critically Ill Patients: A Systematic Review and Meta-Analysis. PLoS ONE.

[208] Wang Z., Jing J., Ren Y., Guo Y., Tao N., Zhou Q., Zhang H., Ma Y., Wang Y. (2019). Preparation and application of selenium nanoparticles in a lateral flow immunoassay for clenbuterol detection. Mater. Lett..

[209] Sonkusre P., Nanduri R., Gupta P., Cameotra S.S. (2014). Improved Extraction of Intracellular Biogenic Selenium Nanoparticles and their Specificity for Cancer Chemoprevention. J. Nanomed. Nanotechnol..

[210] Saif S., Tahir A., Chen Y. (2016). Green Synthesis of Iron Nanoparticles and Their Environmental Applications and Implications. Nanomaterials.

[211] Rajeshkumar S., Bharath L. (2017). Mechanism of plant-mediated synthesis of silver nanoparticles – A review on biomolecules involved, characterisation and antibacterial activity. Chem. Interact..

[212] Sharma D., Kanchi S., Bisetty K. (2019). Biogenic synthesis of nanoparticles: A review. Arab. J. Chem..

[213] Singh P., Kim Y.-J., Zhang D., Yang D.-C. (2016). Biological Synthesis of Nanoparticles from Plants and Microorganisms. Trends Biotechnol..

[214] Zhao X., Zhou L., Rajoka M.S.R., Yan L., Jiang C., Shao D., Zhu J., Shi J., Huang Q., Yang H. (2018). Fungal silver na-noparticles, synthesis, application and challenges. Crit. Rev. Biotechnol..

[215] Habibullah G., Viktorova J., Ruml T. (2021). Current Strategies for Noble Metal Nanoparticle Synthesis. Nanoscale Res. Lett..

[216] Borase H.P., Salunke B.K., Salunkhe R.B., Patil C.D., Hallsworth J.E., Kim B.S., Patil S.V. (2014). Plant extract: A promising bi-omatrix for ecofriendly, controlled synthesis of silver nanoparticles. Appl. Biochem. Biotech..

[217] Govindappa M., Farheen H., Chandrappa C.P., Channabasava, Rai R.V., Raghavendra V.B. (2016). Mycosynthesis of silver nanoparticles using extract of endophytic fungi, Penicillium species of Glycosmis mauritiana, and its antioxidant, antimicrobial, anti-inflammatory and tyrokinase inhibitory activity. Adv. Nat. Sci. Nanosci. Nanotechnol..

[218] Kang X., Kirui A., Muszyński A., Widanage M.C.D., Chen A., Azadi P., Wang P., Mentink-Vigier F., Wang T. (2018). Molecular architecture of fungal cell walls revealed by solid-state NMR. Nat. Commun..

[219] Das S.K., Liang J., Schmidt M., Laffir F., Marsili E. (2012). Biomineralization Mechanism of Gold by Zygomycete Fungi Rhizopous oryzae. ACS Nano.

[220] Mukherjee P., Ahmad A., Mandal D., Senapati S., Sainkar S.R., Khan M.I., Ramani R., Parischa R., Ajayakumar P.V., Alam M. (2001). Bioreduction of AuCl4− ions by the fungus, Verticillium sp. and surface trapping of the gold nanoparticles formed. Angew. Chem. Int. Ed..

[221] Debieux C.M., Dridge E.J., Mueller C.M., Splatt P., Paszkiewicz K., Knight I., Florance H., Love J., Titball R.W., Lewis R.J. (2011). A bacterial process for selenium nanosphere assembly. Proc. Natl. Acad. Sci. USA.

[222] Zhang L., Li D., Gao P. (2012). Expulsion of selenium/protein nanoparticles through vesicle-like structures by Saccharomyces cerevisiae under microaerophilic environment. World J. Microbiol. Biotechnol..

[223] Srivastava S.K., Yamada R., Ogino C., Kondo A. (2013). Biogenic synthesis and characterization of gold nanoparticles by Escherichia coli K12 and its heterogeneous catalysis in degradation of 4-nitrophenol. Nanoscale Res. Lett..

[224] Balakumaran M., Ramachandran R., Balashanmugam P., Mukeshkumar D., Kalaichelvan P. (2016). Mycosynthesis of silver and gold nanoparticles: Optimization, characterization and antimicrobial activity against human pathogens. Microbiol. Res..

[225] Shahverdi A.R., Fakhimi A., Shahverdi H.R., Minaian S. (2007). Synthesis and effect of silver nanoparticles on the antibacterial activity of different antibiotics against Staphylococcus aureus and Escherichia coli. Nanomed. Nanotechnol. Biol. Med..

[226] Prabhu S., Poulose E.K. (2012). Silver nanoparticles: Mechanism of antimicrobial action, synthesis, medical applications, and toxicity effects. Int. Nano Lett..

[227] Krishnaraj C., Jagan E., Rajasekar S., Selvakumar P., Kalaichelvan P., Mohan N. (2010). Synthesis of silver nanoparticles using Acalypha indica leaf extracts and its antibacterial activity against water borne pathogens. Colloids Surf. B Biointerfaces.

[228] Iravani S. (2011). Green synthesis of metal nanoparticles using plants. Green Chem..

[229] Zhang J., Taylor E.W., Wan X., Peng D. (2012). Impact of heat treatment on size, structure, and bioactivity of elemental selenium nanoparticles. Int. J. Nanomed..

[230] Kashyap P.L., Kumar S., Srivastava A.K., Sharma A.K. (2012). Myconanotechnology in agriculture: A perspective. World J. Microbiol. Biotechnol..

[231] Durán N., Marcato P.D., Alves O.L., De Souza G.I.H., Esposito E. (2005). Mechanistic aspects of biosynthesis of silver nanoparticles by several Fusarium oxysporum strains. J. Nanobiotechnol..

[232] Medentsev A., Akimenko V. (1998). Naphthoquinone metabolites of the fungi. Phytochemistry.

[233] Baker R.A., Tatum J.H. (1998). Novel anthraquinones from stationary cultures of Fusarium oxysporum. J. Ferment. Bioeng..

[234] Durán N., Teixeira M.F.S., De Conti R., Esposito E. (2002). Ecological-Friendly Pigments From Fungi. Crit. Rev. Food Sci. Nutr..

[235] Bell A.A., Wheeler M.H., Liu J., Stipanovic R.D., Puckhaber L.S., Orta H. (2003). United States Department of Agricul-ture—Agricultural Research Service studies on polyketide toxins of Fusarium oxysporum f sp vasinfectum: Potential targets for disease control. Pest Manag. Sci. Former. Pestic. Sci..

[236] Jha A.K., Prasad K., Kumar S.A., Thiagarajan S., Wang S.F. (2010). Understanding biosynthesis of metallic/oxide nanoparticles: A biochemical perspective. Biocompatible Nanomaterials Synthesis, Characterization and Applications.

[237] Salunkhe R.B., Patil S.V., Patil C.D., Salunke B.K. (2011). Larvicidal potential of silver nanoparticles synthesized using fungus Cochliobolus lunatus against Aedes aegypti (Linnaeus, 1762) and Anopheles stephensi Liston (Diptera; Culicidae). Parasitol. Res..

[238] El-Seedi H.R., El-Shabasy R.M., Khalifa S.A.M., Saeed A., Shah A., Shah R., Iftikhar F.J., Abdel-Daim M.M., Abdelfatteh O., Hajrahand N.H. (2019). Metal nanoparticles fabricated by green chemistry using natural extracts: Biosynthesis, mechanisms, and applications. RSC Adv..

[239] Mukherjee P., Senapati S., Mandal D., Ahmad A., Khan M.I., Kumar R., Sastry M. (2002). Extracellular synthesis of gold nano-particles by the fungus Fusarium oxysporum. ChemBioChem.

[240] Ahmad A., Mukherjee P., Senapati S., Mandal D., Khan M.I., Kumar R., Sastry M. (2003). Extracellular biosynthesis of silver nanoparticles using the fungus Fusarium oxysporum. Colloids Surf. B Biointerfaces.

[241] Basavaraja S., Balaji S.D., Lagashetty A., Rajasab A.H., Venkataraman A. (2008). Extracellular biosynthesis of silver nanoparticles using the fungus Fusarium semitectum. Mater. Res. Bull..

[242] Andreescu D., Sau T.K., Goia D.V. (2006). Stabilizer-free nanosized gold sols. J. Colloid Interface Sci..

[243] Molnár Z., Bódai V., Szakacs G., Erdélyi B., Fogarassy Z., Sáfrán G., Varga T., Kónya Z., Tóth-Szeles E., Szűcs R. (2018). Green synthesis of gold nanoparticles by thermophilic filamentous fungi. Sci. Rep..

[244] Mukherjee P., Roy M., Mandal B.P., Dey G.K., Mukherjee P.K., Ghatak J., Tyagi A.K., Kale S.P. (2008). Green synthesis of highly stabilized nanocrystalline silver particles by a non-pathogenic and agriculturally important fungus T. asperellum. Nanotechnology.

[245] Sanghi R., Verma P. (2009). Biomimetic synthesis and characterization of protein capped silver nanoparticles. Biores. Technol..

[246] Poluboyarinov P.A., Leshchenko P.P. (2013). A qualitative reaction for cysteine, reduced glutathione, and diacetophenonyl selenide. J. Anal. Chem..

[247] Pankratov A.N., Tsivileva O.M. (2016). In silico Confirmation of the More Active Player Involved in Sulpho-to-seleno Amino Acid Transformations in Mushrooms. Curr. Phys. Chem..

[248] Lloyd J.R. (2003). Microbial reduction of metals and radionuclides. FEMS Microbiol. Rev..

[249] Hansda A., Kumar V. (2016). Anshumali A comparative review towards potential of microbial cells for heavy metal removal with emphasis on biosorption and bioaccumulation. World J. Microbiol. Biotechnol..

[250] Javanbakht V., Alavi S.A., Zilouei H. (2013). Mechanisms of heavy metal removal using microorganisms as biosorbent. Water Sci. Technol..

[251] Perrone G., Susca A., Cozzi G., Ehrlich K., Varga J., Frisvad J., Meijer M., Noonim P., Mahakarnchanakul W., Samson R.A. (2007). Biodiversity of Aspergillus species in some important agricultural products. Stud. Mycol..

[252] Poluboyarinov P.A., Leshchenko P.P., Moiseeva I.Y., Kolesnikova S.G., Epshtein N.B. (2017). Mechanism of reaction of selenium elimination in diacetophenonyl selenide under the action of reduced glutathione. J. Anal. Chem..

[253] Burleson D.J., Driessen M.D., Penn R.L. (2004). On the characterization of environmental nanoparticles. J. Environ. Sci. Health Part A.

[254] Kamnev A., Dyatlova Y., Kenzhegulov O., Vladimirova A., Mamchenkova P., Tugarova A. (2021). Fourier Transform Infrared (FTIR) Spectroscopic Analyses of Microbiological Samples and Biogenic Selenium Nanoparticles of Microbial Origin: Sample Preparation Effects. Molecules.

[255] Tugarova A.V., Mamchenkova P.V., Khanadeev V.A., Kamnev A.A. (2020). Selenite reduction by the rhizobacterium Azospirillum brasilense, synthesis of extracellular selenium nanoparticles and their characterisation. New Biotechnol..

[256] Grønbæk-Thorsen F., Hansen R.H., Østergaard J., Gammelgaard B., Møller L.H. (2021). Analysis of selenium nanoparticles in human plasma by capillary electrophoresis hyphenated to inductively coupled plasma mass spectrometry. Anal. Bioanal. Chem..

[257] Gómez-Gómez B., Sanz-Landaluce J., Pérez-Corona M.T., Madrid Y. (2020). Fate and effect of in-house synthesized tellurium based nanoparticles on bacterial biofilm biomass and architecture. Challenges for nanoparticles characterization in living systems. Sci. Total. Environ..

[258] Bartosiak M., Giersz J., Jankowski K. (2019). Analytical monitoring of selenium nanoparticles green synthesis using photochemical vapor generation coupled with MIP-OES and UV–Vis spectrophotometry. Microchem. J..

[259] Wu S., Sun K., Wang X., Wang D., Wan X., Zhang J. (2013). Protonation of Epigallocatechin-3-gallate (EGCG) Results in Massive Aggregation and Reduced Oral Bioavailability of EGCG-Dispersed Selenium Nanoparticles. J. Agric. Food Chem..

[260] Akçay F.A., Avcı A. (2020). Effects of process conditions and yeast extract on the synthesis of selenium nanoparticles by a novel indigenous isolate Bacillus sp. EKT1 and characterization of nanoparticles. Arch. Microbiol..

[261] Kuroda M., Notaguchi E., Sato A., Yoshioka M., Hasegawa A., Kagami T., Narita T., Yamashita M., Sei K., Soda S. (2011). Characterization of Pseudomonas stutzeri NT-I capable of removing soluble selenium from the aqueous phase under aerobic conditions. J. Biosci. Bioeng..

[262] Zhang H., Zhou H., Bai J., Li Y., Yang J., Ma Q., Qu Y. (2019). Biosynthesis of selenium nanoparticles mediated by fungus Mariannaea sp. HJ and their characterization. Colloids Surf. A Physicochem. Eng. Asp..

[263] Diko C.S., Zhang H., Lian S., Fan S., Li Z., Qu Y. (2020). Optimal synthesis conditions and characterization of selenium nanopar-ticles in Trichoderma sp. WL-Go culture broth. Mater. Chem. Phys..

[264] Rosenfeld C.E., Kenyon J.A., James B.R., Santelli C.M. (2017). Selenium (IV, VI) reduction and tolerance by fungi in an oxic envi-ronment. Geobiology.

